# Combined PET Radiotracer Approach Reveals Insights into Stromal Cell-Induced Metabolic Changes in Pancreatic Cancer In Vitro and In Vivo

**DOI:** 10.3390/cancers16193393

**Published:** 2024-10-04

**Authors:** Alina Doctor, Markus Laube, Sebastian Meister, Oliver C. Kiss, Klaus Kopka, Sandra Hauser, Jens Pietzsch

**Affiliations:** 1 Department of Radiopharmaceutical and Chemical Biology, Helmholtz-Zentrum Dresden-Rossendorf, Institute of Radiopharmaceutical Cancer Research, Bautzner Landstraße 400, 01328 Dresden, Germany; a.doctor@hzdr.de (A.D.); m.laube@hzdr.de (M.L.); s.meister@hzdr.de (S.M.); k.kopka@hzdr.de (K.K.); s.hauser@hzdr.de (S.H.); 2 School of Science, Faculty of Chemistry and Food Chemistry, Technische Universität Dresden, Mommsenstraße 4, 01069 Dresden, Germany; 3 Department of Targetry, Target Chemistry and Radiopharmacy, Helmholtz-Zentrum Dresden-Rossendorf, Institute of Radiopharmaceutical Cancer Research, Bautzner Landstraße 400, 01328 Dresden, Germany; o.kiss@hzdr.de; 4 National Center for Tumor Diseases (NCT) Dresden, Partner Site Dresden, University Cancer Center (UCC), Fetscherstraße 74, 01307 Dresden, Germany; 5 German Cancer Consortium (DKTK), Partner Site Dresden, Fetscherstraße 74, 01307 Dresden, Germany

**Keywords:** tumor microenvironment, radionuclide theranostics, radiotracers, small animal positron emission tomography, spheroids, xenograft models

## Abstract

**Simple Summary:**

Pancreatic cancer is surrounded by a dense fibrotic environment due to the activity of pancreatic stellate cells (PSCs). This hinders the effectiveness of treatment by limiting drug penetration and oxygen delivery. To better understand this, PSCs and cancer cells were studied in laboratory models using radiotracer imaging techniques. The results indicated that while glucose uptake and hypoxia were similar between cancer cells alone and in combination with PSCs, a specific marker of fibrosis (FAPα) was significantly higher in PSC-rich environments. In mice, the marker in question decreased in tumors with only PSCs, indicating that the cells had died. Conversely, the biomarker increased over time in tumors containing both cancer cells and PSCs, suggesting that mouse cells had invaded. This study sheds light on how the tumor environment influences metabolism and thus provides insights for more targeted theranostic applications. In turn, this approach supports the development of more reliable models.

**Abstract:**

**Background/Objective** Pancreatic stellate cells (PSCs) in pancreatic adenocarcinoma (PDAC) are producing extracellular matrix, which promotes the formation of a dense fibrotic microenvironment. This makes PDAC a highly heterogeneous tumor-stroma-driven entity, associated with reduced perfusion, limited oxygen supply, high interstitial fluid pressure, and limited bioavailability of therapeutic agents. **Methods** In this study, spheroid and tumor xenograft models of human PSCs and PanC-1 cells were characterized radiopharmacologically using a combined positron emission tomography (PET) radiotracer approach. [^18^F]FDG, [^18^F]FMISO, and [^18^F]FAPI-74 were employed to monitor metabolic activity, hypoxic metabolic state, and functional expression of fibroblast activation protein alpha (FAPα), a marker of activated PSCs. **Results** In vitro, PanC-1 and multi-cellular tumor spheroids demonstrated comparable glucose uptake and hypoxia, whereas FAPα expression was significantly higher in PSC spheroids. In vivo, glucose uptake as well as the transition to hypoxia were comparable in PanC-1 and multi-cellular xenograft models. In mice injected with PSCs, FAPα expression decreased over a period of four weeks post-injection, which was attributed to the successive death of PSCs. In contrast, FAPα expression increased in both PanC-1 and multi-cellular xenograft models over time due to invasion of mouse fibroblasts. **Conclusion** The presented models are suitable for subsequently characterizing stromal cell-induced metabolic changes in tumors using noninvasive molecular imaging techniques.

## 1. Introduction

Pancreatic ductal adenocarcinoma (PDAC) is one of the deadliest cancers. The 5-year survival rate is only 5% [1,2,3,4]. One of the major challenges in the treatment of PDAC is its resistance to chemotherapy and radiotherapy. In recent years, increasing attention has been paid to the role of pancreatic stellate cells (PSCs) in PDAC progression and therapy resistance. In the healthy pancreas, PSCs are responsible for retinoid storage and for the production and degradation of the extracellular matrix (ECM). In pancreatic cancer, PSCs undergo fibrotic activation, lose their ability to store retinoids, and largely stop degrading the ECM, which promotes the formation of an extensive stromal tumor microenvironment [5,6]. Thereby, PSCs essentially promote the therapy resistance of PDAC [7,8,9,10] PDAC represents a very heterogeneous tumor-stroma entity that is associated with reduced perfusion, restricted oxygen supply, high interstitial fluid pressure, and limited bioavailability of therapeutic agents [7,11]. Factors that contribute to chemoresistance can be physical, such as stiffness and the extracellular matrix acting as a barrier, as well as biological, such as hypoxia, low pH, and cellular interactions [11,12]. Hypoxia is a hallmark of solid cancers and hypoxia-inducible factor-1α (HIF-1α) has been linked to chemoresistance. Strikingly, pancreatic cancer is one of the most hypoxic tumors caused by hypovascularity and desmoplasia, where HIF-1α is found in 88% of patient samples [13,14]. Hypoxia is also contributing to radioresistance, as the presence of oxygen during radiation exposure is necessary for the generation of DNA-damaging free oxygen radicals [15]. Hypoxia is responsible for reactive oxygen species-induced PSC activation, resulting in enhanced ECM production and remodeling and abnormal vascularization [6,7,16,17].

To date, nuclear medicine diagnostics play a minor role in PDAC. Its desmoplastic nature leads to a hypometabolic state, which diminishes the uptake of the standard positron emission tomography (PET) radiotracer [^18^F]FDG (2-[^18^F]fluoro-2-deoxy-D-glucose). From a radiopharmaceutical perspective, new suitable and specific molecular targets and tumor-selective radiotracers are needed to enable better PDAC diagnostics [18,19].

**Figure 1:**
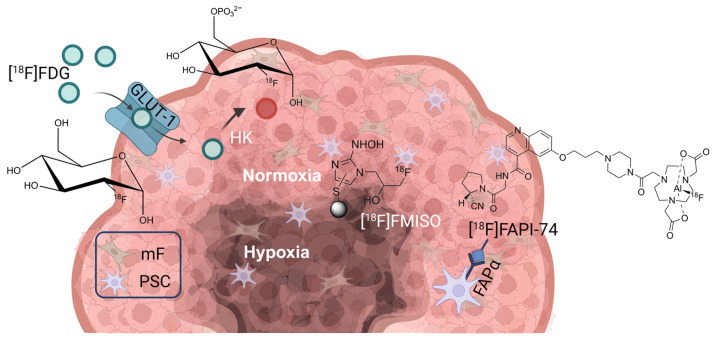
Schematic illustration of the mode of action of the PET radiotracers [^18^F]FDG, [^18^F]FMISO (fluoromisonidazole), and [^18^F]FAPI-74. In addition to tumor cells, the tumor shown here also consists of PSCs and mouse fibroblasts (mF). The tumor consists of a metabolically active and normoxic marginal zone (pink), while the inner core represents a hypoxic region (brown). [^18^F]FDG (cyan circles) is taken up by the glucose transporter 1 (GLUT-1) transporter and metabolized by hexokinases (HK), then becoming trapped (red circle) in metabolically active regions of the tumor. [^18^F]FMISO binds to macromolecules (blue circle) in the hypoxic region. To target the tumor stroma, [^18^F]FAPI-74 can be used. The tracer binds to FAPα in cancer-associated fibroblasts. Created in BioRender. Doctor, A. (2024) BioRender.com/b29q105.

Recently, the targeting of fibroblast activation protein alpha (FAPα) offered an interesting approach [20]. FAPα is a membrane-bound type 2 serine protease [21] and is highly expressed by cancer-associated fibroblasts (CAFs) [22], but only in low levels by tumor cells or normal tissue [23,24]. Addressing FAPα offers a novel approach for tumor imaging by focusing on tumor-associated cells (see Figure 1). This requires models that reflect the coexistent microenvironment situation, specific to the tumor entity and its associated stromal cells. For PDAC, a co-cultured model comprising of PSCs and PDAC cells is particularly promising. Co-culture spheroids and xenografts are referred to herein as multicellular tumor spheroids (MCTS) and multi-cellular xenografts, respectively.

Developing reliable in vitro and in vivo models that closely mirror the clinical situation is essential for effective radiotracer or radiotherapeutics development. In vitro, 3D models, particularly tumor spheroids, better recapitulate factors such as tumor microenvironment and cell interactions than monolayer cultures conducted [25,26,27,28]. The influence of physicochemical and pathophysiological parameters such as interstitial pressure, pH, and hypoxic and necrotic regions can be reliably modeled. This allows investigations on tissue perfusion, specific and non-specific radiotracer uptake, and locoregional accumulation of new radiotracers [27]. The next step is to translate the 3D cell model approach to a preclinical in vivo model. From a radiopharmaceutical perspective, optimal models not only reflect the physiological situation of the respective tumor entity but also deliver tumor lesions of a defined size at a specific time point. For the radiopharmacological characterization of new tracers, it is crucial to reliably distinguish tumors from normal tissue. Tumor localization should avoid superimposition of tumor-accumulated activity with activity in metabolically active organs such as the liver, gallbladder, intestine, kidneys, and urinary bladder. Last but not least, human xenograft tumors expressing the human molecular targets are the first choice to evaluate potential radiotracers.

In this study, we established and characterized new models for radiopharmacological studies of tracer by implementing PSCs in both 3D in vitro and subcutaneous xenograft PDAC models.

## 2. Materials and Methods

### 2.1. Cell Culture

Pancreatic ductal cancer cell line PanC-1 was purchased from ATCC (CRL-1469). Pancreatic stellate cells (HPaSteC) were purchased from ScienCell (Carlsbad, California, USA) (#3830). PanC-1 cells were cultured in Dulbecco’s Modified Eagle Medium (DMEM) supplemented with 10% fetal calf serum (FCS) (F7524, Sigma-Aldrich, St. Louis, Missouri, USA) and 1% Penicillin/Streptomycin solution (Biochrom, Berlin, Germany). HPaSteC cells were cultured in SteCM media supplemented with 2% of fetal bovine serum (ScienCell, Cat. No. 0010), 1% of stellate cell growth supplement (SteCGS, ScienCell, Cat. No. 5352), and 1% of antibiotic solution (ScienCell, Cat. No. 0503). Cell lines were routinely analyzed by PCR for mycoplasma contamination. Cell incubation was conducted under standard cell culture conditions (37 °C, 5% CO_2_, 95% humidity).

HAP1 (C631) and HAP1PLOD2knock-out (HZGHC005475c004) cells were purchased from Horizon Discovery and cultured according to manufacturer guidelines.

### 2.2. Generation of Spheroids

Spheroids were cultured as described elsewhere, using methylcellulose [29]. For multi-cellular spheroids, a total cell number of 16,000 cells/spheroid was maintained, and PanC-1 and HPaSteC were mixed ahead of seeding in a 1:3 ratio in SteCM media. In accordance with the existing literature on this subject, which indicates that a ratio of 1:3 is of biological significance [30,31], we selected a ratio of 1:3 for our experiments. For determining the effect of extrinsic hypoxia, spheroids were cultivated for 7 days either under normoxic (37 °C, 5% CO_2_, 21% O_2_, 95% humidity) or hypoxic (37 °C, 5% CO_2_, 1% O_2_, 95% humidity) conditions.

### 2.3. Radiosynthesis of [^18^F]FAPI-74

The radiosynthesis of [^18^F]AlF-FAPI-74, hereafter referred to as [^18^F]FAPI-74, was optimized and automated as detailed in the Supplementary Information Figures S8–S12 and Table S2. The FAPI-74 precursor was obtained from SOFIE (SOFIE Biosciences, Dulles, VA, USA).

### 2.4. Radiotracer Uptake In Vitro

For in vitro radiotracer uptake experiments, growth medium was removed and replaced by medium mixed with PET tracers [^18^F]FDG and [^18^F]FMISO [32] as well as [^18^F]FAPI-74 [33], corresponding to 0.1–0.2 MBq per well. For [^18^F]FDG experiments, glucose-free medium was used. Spheroids were incubated for 5, 10, 30, 60, and 120 min in the case of [^18^F]FDG, 4 h with [^18^F]FMISO, or 30 min with [^18^F]FAPI-74. The spheroids were washed three times with cold PBS containing 0.9 mol/L CaCl_2_ and 0.5 mol/L MgCl_2_. The amount of radiotracer in the spheroids was determined by measuring counts per minute using a gamma counter (Wizard2 PerkinElmer, Waltham, Massachusetts, USA), and the decay-corrected data were normalized to the spheroid protein content established via Bicinchoninic acid assay.

### 2.5. Animal Experiments

All animal experiments were carried out according to the guidelines of the German Regulations for Animal Welfare and have been approved by the local Ethical Committee for Animal Experiments (reference number DD24.1-5131/449/49). For the xenograft mouse models, SCID (severe combined immunodeficiency disease) mice were subcutaneously injected with 5 × 10^6^ cells suspended in 100 μL phosphate-buffered saline (PBS) in their right leg. The cell suspension consisted of either PanC-1 cells or a mixture of PanC-1 and HPaSteC cells in a 1:3 ratio. The welfare of the mice was monitored daily, and their weight and tumor growth were measured and recorded three times a week. Tumor size was determined by caliper, and tumor volume was calculated assuming a tri-axial ellipsoid with the axes a, b, and c using the formula V = π/6 × abc.

### 2.6. Small Animal PET/CT

Mice were anesthetized by inhalation of 10% (*v*/*v*) desflurane in 30/70% (*v*/*v*) oxygen/air and were continuously warmed to 37 °C. Small animal PET was performed using the nanoPET/CT scanner (Mediso Medical Imaging Systems, Budapest, Hungary). Animals received intravenous application of [^18^F]FDG or [^18^F]FAPI-74 corresponding to 15 MBq per mouse through a tail vein catheter and were measured dynamically for 1 h. 24 h after the PET/CT with [^18^F]FDG, the mice were anesthetized again and intravenously injected with 15–20 MBq of [^18^F]FMISO and subjected to 30 min static PET/CT measurements at 4 h p.i. Subsequently, the mice were intravenously injected with 60 mg/kg pimonidazole and 5 μL/g Hoechst 33,342 and sacrificed 90 min after pimonidazole and 1 min after Hoechst application by cervical dislocation. The tumor was removed and stored for analysis.

With each PET scan, a corresponding computed tomography (CT) image was recorded and used for anatomical referencing and attenuation correction. For binning, framing and image reconstruction protocols described elsewhere were used [34,35]. The three-dimensional list-mode data were binned using the 400–600 keV energy window and sorted in up to 36 time frames. The Tera-TomoTM 3D algorithm was used to reconstruct the time frames with corrections for decay, scatter, and attenuation and a voxel size of 0.4 mm. Images were post-processed and analyzed using ROVER (ABX GmbH, Radeberg, Germany) and displayed as maximum intensity projections (MIPs) at indicated time points and scaling. The time points displayed in graphs represent the mid-frame timepoints. Three-dimensional volumes of interest (VOIs) were defined using a fixed threshold of 39% for delineation of the tumor. Standardized uptake values (SUV) were determined and reported as SUVmean (VOI-averaged) and SUVmax (VOI-maximum). Time-activity curves were generated for tumor VOIs and further analyzed using Prism 10.3.0 (GraphPad Software, San Diego, CA, USA).

### 2.7. MRI

Additional to PET/CT measurements, xenograft mice were measured using dedicated 7T small animal magnetic resonance imaging (MRI) BioSpec USR 70/30 (Bruker BioSpin GmbH & Co. KG, Ettlingen, Germany) and ParaVision software (version 6.0.1) with a T2-weighted measuring sequence (TRARE) as previously described elsewhere [36]. A repetition time of 2476 ms and an echo time of 39 ms were used with a spatial resolution of 75 µm in the xy direction and a slice thickness of 0.5 mm. In addition, a diffusion-weighted image sequence (DWI) was recorded with diffusion encoding constants (b) of 5, 10, 25, 50, 75, 100, 125, 150, 175, 200, 500, 750, and 1000 s/mm^2^. A diffusion gradient duration of 3.65 ms and a diffusion separation time of 11.6 ms were used. Apparent diffusion coefficient (ADC) values were calculated by ParaVision software (version 6.0.1).

### 2.8. Immunohistochemistry

Specific tissue responses were visualized using immunohistological staining for multiple cellular markers. Formalin-fixed and paraffin-embedded tumor sections were rehydrated using Roticlear (Carl Roth, Karlsruhe, Germany) and a graded series of ethanol. Antigen retrieval was performed in 10 mmol/L citrate buffer pH 6 heated to 95 °C for 20 min using a steamer. The slides were washed with 0.05 mol/L Tris-buffered saline pH 8 containing 0.5% (*v*/*v*) Tween-20 (TBS-T). Endogenous peroxidase was quenched using 3% H_2_O_2_ in TBS-T, and endogenous avidin and biotin blocking was performed (Agilent, Santa Clara, CA, USA). Non-specific binding sites were blocked using 10% fetal bovine serum (*v*/*v*) in TBS-T. Afterward, tissue sections were incubated overnight with the primary antibodies summarized in Supporting Information Table S1. HP3-100 Kit rabbit antisera Hypoxyprobe Omni Kit Pab2627 for pimonidazole staining. The next day, sections were incubated with biotinylated secondary antibody for 1 h and visualized with ExtrAvidin-peroxidase E2886 (Sigma-Aldrich, St. Louis, MO, USA), followed by staining with an AEC-staining kit (BD Biosciences, Eysins, Switzerland). Sections were counterstained with Meyer’s hematoxylin and mounted with Kaiser’s glycerol gelatin (Carl Roth). The AXIO Imager A1 microscope (Carl Zeiss, Oberkochen, Germany) was used for imaging. Quantification of immunohistochemical staining was performed using ImageJ/FIJI (version 2.14.0). A color threshold plug-in was used and set for immunohistochemically positive stained areas. The positively stained area was analyzed per 1 mm^2^ of tumor section. For hematoxylin and eosin (H&E), staining sections were stained for 3 min in Mayer’s hematoxylin, followed by staining for 30 s in eosin. Sections were then washed in a graded series of ethanol.

### 2.9. SDS-Page and Western Blotting

FAPα was detected by SDS-PAGE followed by Western blotting as reported elsewhere [37].

The membrane was probed with the primary antibody for FAPα (ab207178, Abcam, Cambridge, United Kingdom) and anti-β-actin (ab5316, Abcam, Germany) and with the corresponding secondary antibody conjugated to horseradish peroxidase (A0545, Sigma-Aldrich, Germany). Finally, the proteins were visualized by chemiluminescence using SuperSignal^®^ West Pico and Femto Chemiluminescent Substrate (Thermo Fisher Scientific, Waltham, Massachusetts, USA) and imaged using a MF-ChemiBIS Bio-Imaging System (Biostep GmbH, Jahnsdorf, Germany).

### 2.10. Statistical Analysis

Statistical analysis was conducted using Prism 10.1.2 (GraphPad Software, San Diego, CA, USA). Differences were tested for significance using ANOVA and the Tukey post-hoc test. Significance was considered at *p*-values < 0.05.

## 3. Results

### 3.1. Characterization of Spheroid Models

**Figure 2:**
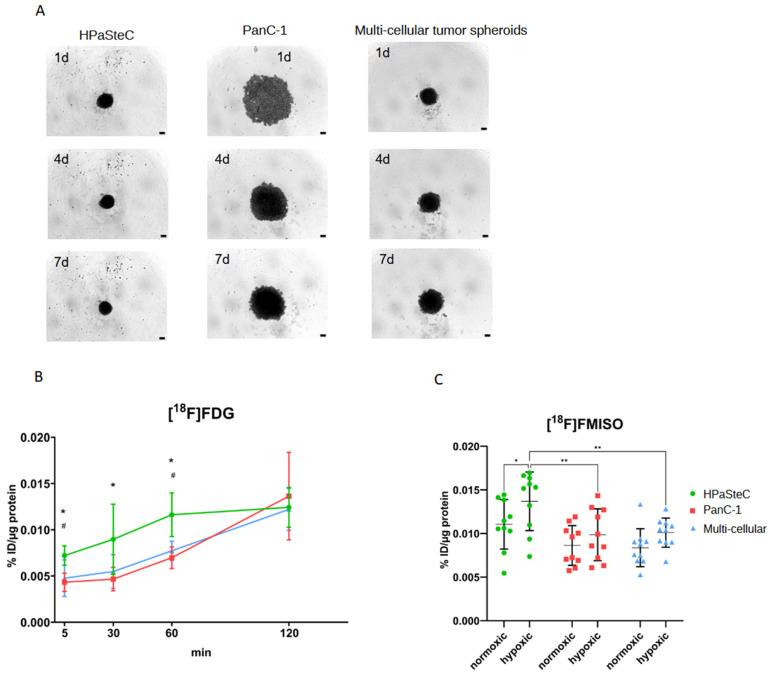
Characterization of spheroid models. (**A**) Representative spheroid images of HPaSteC, PanC-1, and MCTS after 1, 4, and 7 days of incubation. Scale bar corresponds to 100 µm. (**B**,**C**) In vitro radiotracer uptake assay with HPaSteC, PanC-1, and MCTS spheroids. Percentage of injected dose per µg (mean + SD) and statistical difference *: HPaSteC vs. PanC-1, #: HPaSteC vs. MCTS (*p* < 0.05, two-way ANOVA). (**B**) time-dependent [^18^F]FDG uptake. (**C**) [^18^F]FMISO uptake in normoxic and hypoxic conditions after 4 h of incubation. Statistical difference (* *p* < 0.05, ** *p* < 0.0021 two-way ANOVA).

The starting point for subsequent model characterization was the cultivation of spheroids. As shown in Figure 2A, HPaSteC spheroids were smaller than PanC-1 spheroids with the same initial cell number. Additionally, spheroids did not increase in size over time but rather became denser, as observed through confocal microscopy. MCTS were also smaller and had a denser structure than the PanC-1 spheroids.

The uptake of [^18^F]FDG by spheroids was measured at different time points after application (see Figure 2B). HPaSteC spheroids exhibited significantly higher uptake of [^18^F]FDG at time points 5 to 60 min compared to PanC-1 spheroids and at 5 and 60 min compared to MCTS. The uptake of [^18^F]FMISO was evaluated under normoxia and external hypoxia (see Figure 2C). Hypoxic atmospheric conditions resulted in a significantly higher accumulation of [^18^F]FMISO tracer in HPaSteC spheroids in comparison to PanC-1 and MCTS. Upon comparison of the two conditions, significant higher accumulations of [^18^F]FMISO were evident in the case of HPaSteC spheroids cultured in hypoxic environments compared to normoxic conditions. In contrast, no effects were observed for PanC-1 spheroids and MCTS. Similar to our observations with [^18^F]FDG, the MCTS exhibited comparable uptake values to those of PanC-1 cells, despite containing three-quarters HPaSteC cells and only one-quarter PanC-1 cells. Similar results were obtained using a monolayer model (see Supporting Information Figure S1).

### 3.2. Growth and Radiopharmaceutical Characterization of Xenograft Tumors

**Figure 3:**
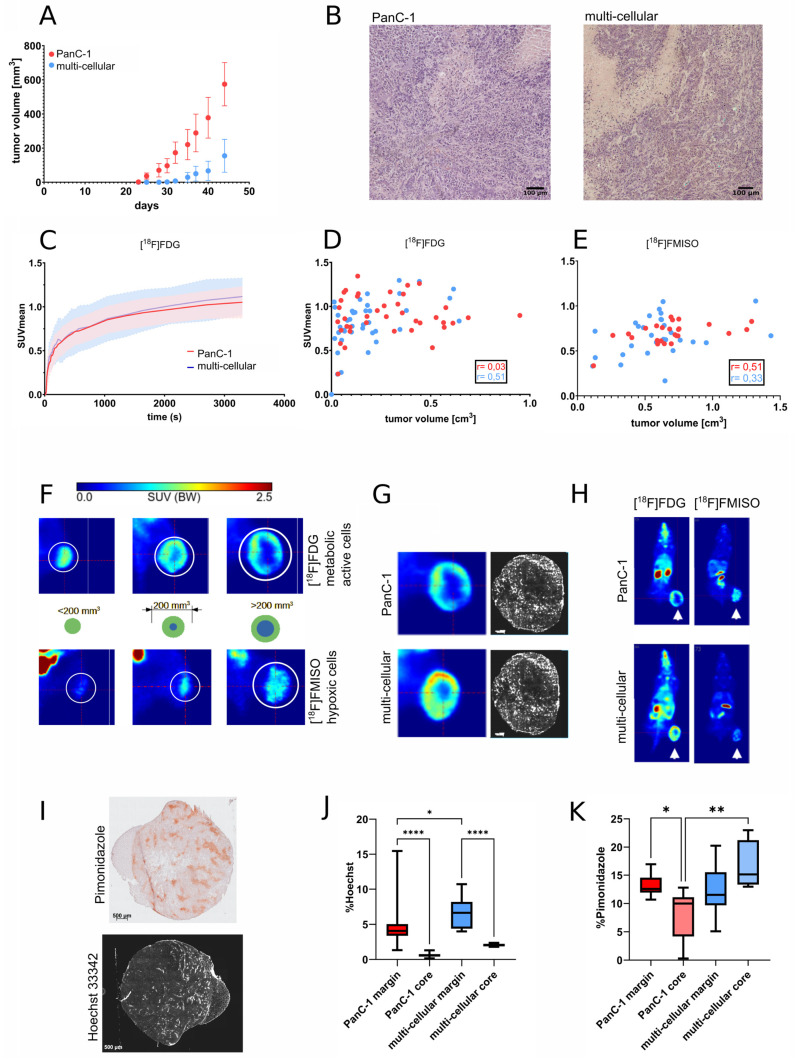
Characteristics of PanC-1 and multi-cellular xenografts. (**A**) Growth curve of subcutaneous tumors in SCID beige mice after injection of PanC-1 cells and multi-cellular xenografts (mean ± SD, n = 15). (**B**) Representative histological images of H&E staining. (**C**) Dynamic PET measurements of [^18^F]FDG. (**D**) PET measurements depicting the SUVmean of [^18^F]FDG and (**E**) [^18^F]FMISO in PanC-1 and multi-cellular tumor-bearing SCID mice versus tumor volume in cm^3^ and Pearson’s correlation (r). (**F**) Illustration of size-dependent core formation. [^18^F]FDG PET showing metabolically active tumor regions (**top row**) and [^18^F]FMISO PET in the same mouse showing hypoxic tumor microenvironment (**bottom row**). The middle row illustrates the tumor size-dependent extent of metabolically active rim and hypoxic core. (**G**) Exemplary comparison between [^18^F]FDG PET imaging and fluorescent dye Hoechst 33342 for vascularization imaging. For better visibility, the blue channel was changed to white. (**H**) Sequential [^18^F]FDG and [^18^F]FMISO PET images of mice bearing xenograft tumors (white arrows). (**I**) Representative image of Hoechst 33342 fluorescence for blood vessel staining and pimonidazole staining for hypoxia. (**J**) Percentage of pimonidazole-positive tumor area and Hoechst fluorescence (**K**) in the tumor margin and core. Statistical analysis with one-way ANOVA (*p* * 0.0332, ** 0.0021, **** < 0.0001).

The xenograft multi-cellular model was used to determine whether the similarities in radiotracer uptake observed in vitro between MCTS and cancer cells could be observed in vivo. Either PanC-1 cells alone or a cell mixture of PanC-1 and HPaSteC were injected into SCID mice. Tumor growth was observed 23 days after injection for PanC-1 and 30 days for multi-cellular tumors (see Figure 3A). Throughout the experiment, PanC-1 tumors consistently grew faster than multi-cellular tumors. Regarding tumor morphology, multi-cellular tumors exhibited a more uneven and knobbly appearance compared to PanC-1 tumors. Moreover, a hematoxylin and eosin (H&E) overview staining of the tumor samples was accomplished to analyze the morphologic organization (Figure 3B). Extensive deposition of connective tissue was observed in both models.

In parallel to the approach of injecting a mixture of HPaSteC and PanC-1 cells, an approach of injecting a mixture of two other fibroblast-like cell lines, HAP1 and HAP-1/PLOD2kockout, with PanC-1 cells was investigated. These cells are human haploid cell lines with fibroblast-like properties. HAP1 represents the wild type, while HAP-1/PLOD2kockout cells lack PLOD2 (Procollagen-lysine,2-oxoglutarate 5-dioxygenase 2), an enzyme involved in extracellular matrix formation [38]. It should be noted that not all experiments were conducted in these two additional models, and thus a direct comparison cannot be made here. However, the findings from these models support the results reported in the primary model approach. Additional studies on these models are exclusively reported in the Supplementary Materials Section Figures S2 and S3.

Uptake of [^18^F]FDG and [^18^F]FMISO was investigated in dependence of time (Figure 3C) and tumor volume. Figure 3D shows the SUVmean of [^18^F]FDG corresponding to the tumor size for PanC-1 as well as multi-cellular xenograft tumors. There is a steep increase in [^18^F]FDG uptake followed by a constant uptake for tumors larger than 0.2 cm^3^. A gradual increase of [^18^F]FMISO uptake with increasing tumor volume is observed in both tumor models (Figure 3E). Upon comparison of [^18^F]FDG and [^18^F]FMISO images of corresponding tumors, it became evident that there is a size-dependent change of local tracer accumulation. Tumors smaller than 0.2 cm^3^ exhibit a homogeneous [^18^F]FDG uptake, while larger tumors display a distinct [^18^F]FDG uptake in the marginal zone of the tumor along with a core. The core, as seen through [^18^F]FDG imaging, is also visible as a counterpart through [^18^F]FMISO imaging (Figure 3F). In large tumors, the core is already pronounced, and the [^18^F]FMISO accumulation is extensive. The formation of a hypoxic core in our model is independent of the tumor’s composition (Figure 3H). The tissue distribution of injected Hoechst 33342 was used to visualize the vascularization of the tumor. Blood vessels were mainly detected in the outer zone of the tumor, with the inner part showing only sparse distribution of blood vessels. This less vascularized inner part of the tumor corresponds in size and shape to the core visible in PET imaging in the same mouse (Figure 3G).

To confirm our results, ex vivo investigations using pimonidazole as a hypoxia marker and semi-quantitative analysis of vascular distribution were conducted. Tumor sections were analyzed by dividing the tumor into a marginal zone and an inner core. Hoechst 33342 and pimonidazole staining were imaged by fluorescence microscopy and bright field microscopy, respectively (Figure 3I). The percentage of fluorescence or staining per 1 mm^2^ tumor section was calculated. The results in Figure 3J show that in both tumors, the marginal zone is better vascularized, as indicated by significantly higher Hoechst fluorescence compared to the core zone. In addition, multi-cellular tumors have a significantly higher percentage of Hoechst fluorescence in the marginal zone compared to PanC-1 tumors. For pimonidazole staining (Figure 3K), significant differences were observed for PanC-1 tumors between the marginal zone and the core. Although there is no significant difference in the percentage of pimonidazole staining between PanC-1 and multi-cellular tumor margin, the multi-cellular tumor core is significantly more stained than the core of PanC-1 tumors, corresponding to a higher degree of hypoxia.

### 3.3. MRI Imaging

**Figure 4:**
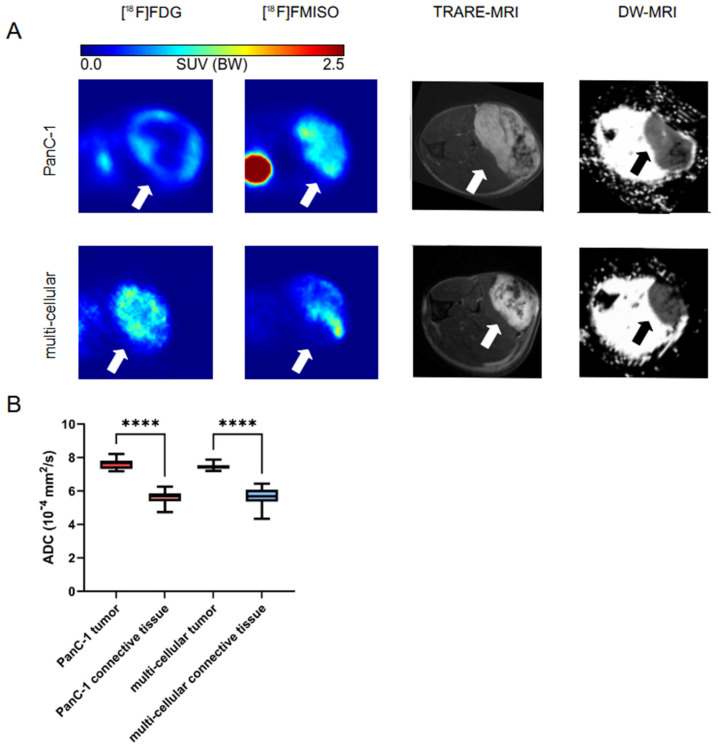
PET and MRI imaging reflect hypoxia and connective tissue. (**A**) Sequential [^18^F]FDG and [^18^F]FMISO PET images of mice bearing xenograft tumors in comparison of T2 weighted MRI (TRARE) and diffusion weighted MRI (DW) images. Arrows indicating the tumor lesion. (**B**) ADC values calculated for DW-MRI images with significant differences of **** *p* < 0.0001 (One-way ANOVA).

An MRI scan was conducted prior to PET imaging, utilizing both a turbo rapid acquisition with relaxation enhancement (TRARE) and a diffusion-weighted (DW) sequence (Figure 4). In DW-MRI, the diffusion strength is measured rather than the orientation. Structures exhibiting high diffusion appear hypointense, while those exhibiting reduced diffusion demonstrate hyperintensity signals. The dark areas observed in the tumor region in TRARE-MRI are similarly visible in DW-MRI. The ADC of these regions was calculated to be 5.6 × 10^−4^ mm^2^/s for both tumor models, while the remaining tumor tissue was calculated to be 7.6 and 7.4 × 10^−4^ mm^2^/s for PanC-1 and multi-cellular tumors, respectively. Significant differences in apparent diffusion coefficient (ADC) were observed between connective tissue and remaining tumor tissue for both tumor models.

### 3.4. FAPα as Marker of PSCs

So far, only marginal differences have been observed between PanC-1 and MCTS, as well as the two xenografts in [^18^F]FDG and [^18^F]FMISO accumulation. This finding is intriguing, as it is widely agreed that pancreatic stellate cells play a critical role in tumorigenesis and stroma formation. Therefore, we wanted to further investigate why our models did not show a significant difference in terms of glucose metabolism and hypoxia. One possibility is that the PSCs were dead or overgrown at the time of imaging due to the long tumor growth time of approximately two to three months. Consequently, a tracer was needed that could specifically image PSCs.

It is known that PSCs express the FAPα [21,39,40], and thus, we used FAPα as a selective marker to show the presence or decline of human PSCs.

**Figure 5:**
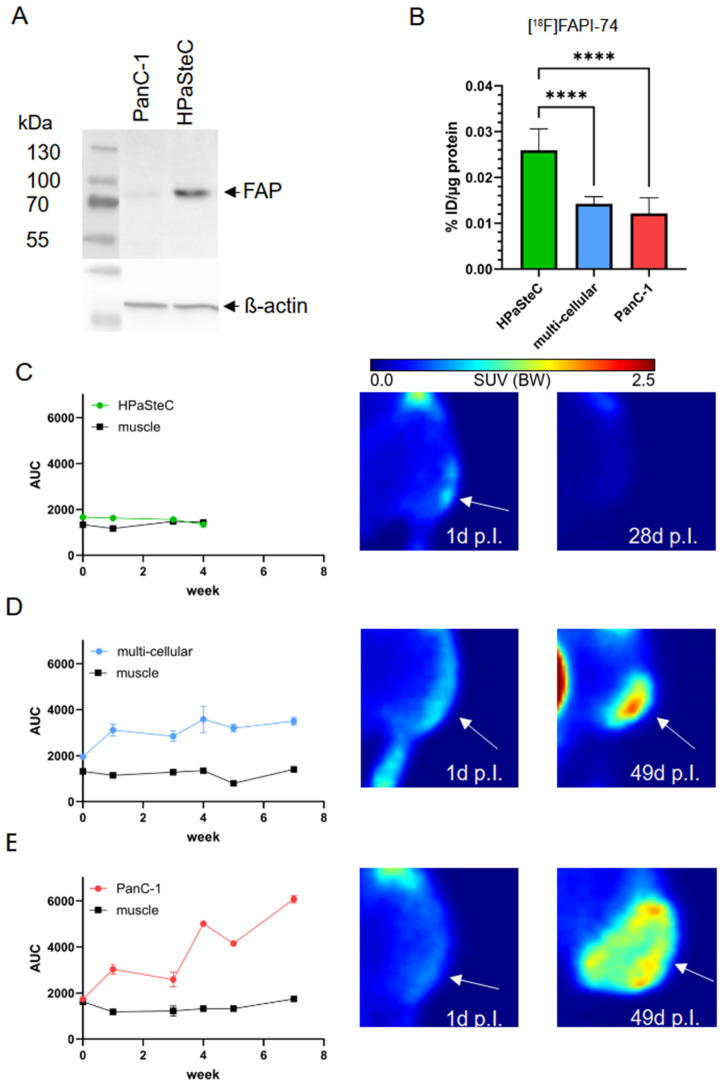
[^18^F]FAPI-74 as a PSC marker. (**A**) FAPα expression was determined via Western blot using cell lysates. (**B**) [^18^F]FAPI-74 uptake by spheroids after 30 min. Statistical analysis with one-way ANOVA (**** *p* < 0.0001, n = 15). In vivo [^18^F]FAPI-74 uptake in HPaSteC (**C**), multi-cellular (**D**), and PanC-1 (**E**) xenograft tumor shown as Area under curve (AUC) in the course of 7 weeks and the corresponding PET images to the indicated time points after injection. The white arrow indicates the injection site. The original Western blot figures can be found in Supplemental Materials Figure S6.

Western blot analysis revealed expression of FAPα in HPaSteC cells, whereas its expression in PanC-1 cells was scarce (Figure 5A, Supplementary Figure S6). In vitro, [^18^F]FAPI-74 uptake in spheroids confirmed significantly lower uptake in PanC-1 spheroids compared to MCTS and HPaSteC spheroids (Figure 5B). This confirms FAPα as a suitable marker for PSCs. Experiments with HAP1 and HAP1/PLOD2 knock-out spheroids demonstrated a significant higher uptake in mixture with PanC-1 for both cell lines, as well as a higher uptake compared with PanC-1 (see Supporting Information Figure S2).

[^18^F]FAPI-74 PET/CT in xenograft-bearing mice was conducted 1 day, as well as 1, 3, 4, 5, and 7 weeks after cell injection to detect the presence of FAPα in the tumors (Figure 5C–E).

PSCs do not form tumors; however, initial uptake directly at the cell injection site after intravenous [^18^F]FAPI-74 injection was observed. During the three-week period, the area under curve (AUC) decreased and was undetectable about 4 weeks after injection (see Figure 5C). Consequently, the AUC for the tumor was corresponding to the AUC of the muscle at 4 weeks after injection, indicating no difference in SUV between the tumor and muscle (Figure S4). This suggests that PSCs died successively over four weeks.

However, PanC-1 and multi-cellular tumors showed an increase in AUC at each time point until the end of the 49-day experiment (see Figure 5D,E).

### 3.5. Immunohistological Staining

**Figure 6:**
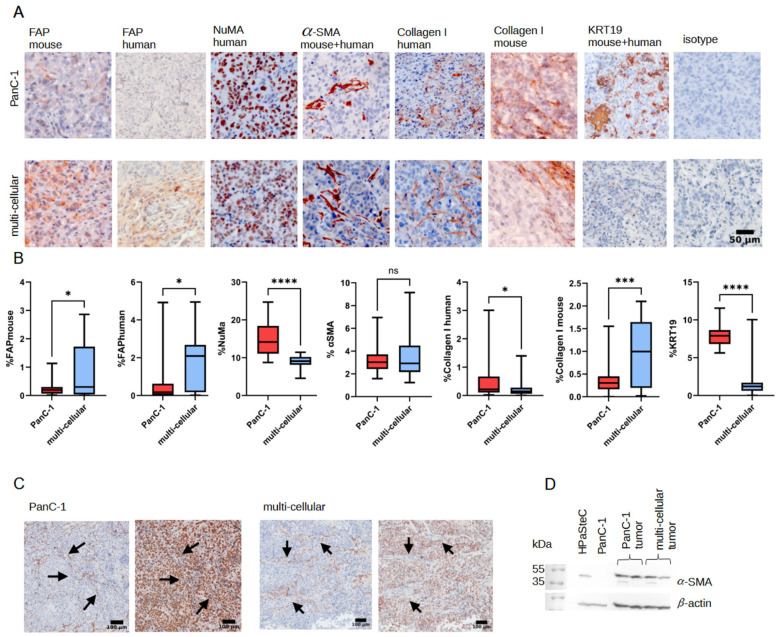
(**A**) Representative immunohistological images of markers for murine and human FAP, human nuclear mitotic antigen (NuMa), α-SMA (smooth muscle actin), human and murine Collagen I, and Cytokeratin 19 (KRT19). Staining control was performed using rabbit isotype antibody. Hematoxylin counterstains the cell nuclei in blue, and positive immunohistological staining is red. (**B**) Quantitative analysis of immunohistological positive staining with ImageJ (significant differences calculated with *t*-test *p* < 0.05, * 0.0332, *** 0.0002, **** <0.0001). (**C**) Sequential immunohistological staining of α-SMA and human cell nuclei in multi-cellular and PanC-1 xenograft tumors. Black arrows indicate blue nuclei corresponding to positive α-SMA staining. (**D**) Western blot shows α-SMA protein expression in monolayer cultured HPaSteC and in xenograft PanC-1 and multi-cellular tumors and none in PanC-1 cells. β-actin was used as loading control. The original Western blot figures can be found in Supplemental Materials Figure S7.

To investigate the increase in [^18^F]FAPI-74 uptake in PanC-1 and multi-cellular tumors, immunohistological staining on both xenograft tumor models was performed. Figure 6 shows extensive positive staining for α-SMA in both models. NuMA was used as a marker for human cells. Tumors infiltrated by mouse cells were observed as early as 12 days post-injection (see Supporting Information Figure S5). The detection of α-SMA and NuMa in sequential tumor sections revealed that tumor-infiltrating mouse cells are α-SMA-positive. PanC-1 cells in monolayer are α-SMA-negative, as confirmed by western blotting (Figure 6C). Therefore, it is assumed that the positive staining of α-SMA in PanC-1 tumors, as well as in western blot, is a result of α-SMA-expressing mouse cells. Immunohistochemistry revealed mouse collagen I in addition to human collagen I in PanC-1 as well as multi-cellular tumor sections. Additionally, the tumor sections were also positive for murine FAPα. Analysis of positive immunohistological staining revealed significant differences between the two tumor models. While PanC-1 tumors showed significantly more positive staining for human cells and human collagen I as well as KRT19, multi-cellular tumors showed significantly more positive staining for human as well as mouse FAPα as well as mouse collagen I.

When culturing PanC-1 tumors with HAP1 and HAP1 PLOD2-knockout cells, positive α-SMA and human Collagen I staining was observed, too, along with mouse cells that were visualized via NuMA staining (see Supplementary Information).

## 4. Discussion

In this study, we present a combined radiotracer approach for radiopharmacological characterization of MCTS and tumor xenograft models of PDAC in vitro and in vivo. Therefore, a human PDAC cell line and human pancreatic stellate cells were used as examples. A supporting approach was carried out in parallel using another fibroblast-like human cell line (HAP-1). This model system allows the investigation of potential radiotracers targeting either tumor cells or, as shown here, specifically relevant stromal cells. Moreover, our combined radiotracer approach revealed stromal cell-induced metabolic changes. In addition, this approach enables us to better assess the quality of the models used and supports the development of more reliable models.

During growth, spheroids can develop tumor-like characteristics, including nutrient gradients and hypoxia [27,41,42,43]. MCTS of two or more cell types offer a more realistic model for studying cellular interactions and signaling [27]. Previous studies have demonstrated the efficacy of MCTS with PSCs and PDAC cells as a model for studying chemosensitivity [44], immunosuppression [45], and for investigating tumor and stromal cell crosstalk [46].

We used healthy human pancreatic stellate cells (HPaSteC), which caused an increase in migration and proliferation as well as chemotherapy resistance of cancer cells in previous studies. These effects have not been induced by immortalized or murine PSCs [47]. HPaSteC spheroids exhibited a dense structure and pronounced hypoxia that well reflect the clinical situation, in which PSCs are responsible for a dense stromal environment that restricts blood flow and therefore oxygen and drug accessibility to the tumor. Interestingly, hypoxia and glucose uptake of MCTS were more similar to those of monoculture spheroids of pancreatic cancer cells, although the seeded cell number was composed of three-quarters of stellate cells. This indicates that hypoxia and glucose uptake were altered by cellular interactions of cancer and stellate cells within the spheroids. A significantly higher [^18^F]FMISO accumulation under external hypoxia compared to normoxia was observed for HPaSteC spheroids only. This can be attributed to the dense structure of HPaSteC spheroids, which remain hypoxic despite the normoxic flush when harvesting the spheroids at the end of the experiment.

As the next step, we established a complementary subcutaneous xenograft model based on the same cell types in immunodeficient mice.

In contrast to available literature, we observed PanC-1 tumors to be significantly larger than multi-cellular tumors. For example, Vonlaufen et al. detected an increase in tumor size in PDAC+PSC orthotopic xenografts [48]. They used 1 million PSCs in addition to 1 million pancreatic cancer cells, thereby doubling the total cell number. We avoided the possibility that mixed tumors are simply larger because of the higher cell number by keeping the total cell number the same. PanC-1 tumors were three to four times larger than multi-cellular tumors, which corresponds to the difference in tumor cell number injected.

Referring to the altered metabolic activity and hypoxia observed in MCTS, we investigated whether tumor formation from PDAC and PSC cells had a comparable effect in the xenograft model.

Unexpectedly, glucose uptake remained constant after tumors exceeded a volume of approximately 0.2 cm^3^. This effect was independent of the cellular composition of the tumors. [^18^F]FMISO imaging revealed the formation of a hypoxic core in tumors of 0.2 cm^3^ or larger, without evidence of necrosis. In these tumors, the fraction of metabolically active cells that consumed [^18^F]FDG remained constant, whereas the fraction of hypoxic cells that accumulated [^18^F]FMISO increased. This provides an explanation as to why there is no size-dependent increase in [^18^F]FDG accumulation in tumors larger than 0.2 cm^3^. This recapitulates the clinical situation, where PDAC is described as a hypoxic tumor entity due to its desmoplastic nature [13,49,50]. Quantity and organization of blood vessels is a major determinant of hypoxia. Analysis of margin and core zones in tumor sections revealed a higher degree of vascularization in the margin than in the core zones of both PanC-1 and multi-cellular tumors, with this difference being significantly greater in multi-cellular tumors. It can thus be concluded that the observed hypoxia in the tumor core is the consequence of inadequate vascularization.

This underlines the proangiogenic functions of PSCs [51,52]. Despite the higher degree of vascularization, hypoxia was higher in the core region of multi-cellular tumors.

Based on multiple publications emphasizing the importance of PSCs in tumors [5,6,7,52,53,54,55,56,57,58], we hypothesized that HPaSteC would increase the stroma formation in multi-cellular tumors and thereby also affect hypoxia and glucose turnover. However, DW-MRI revealed that the presence of connective tissue was comparable in both tumor models, as indicated by the dark regions (Figure 4A) observed. The ADC of these regions was found to be significantly lower than in the remaining tumor tissue. This finding is consistent with those of Hauge et al. and Wegner et al., who demonstrated that areas with a high collagen content exhibited a correlation with low ADC values [59,60]. Magnetic resonance imaging is unable to distinguish between human and mouse collagen. However, immunohistochemical staining demonstrated that PanC-1 tumors exhibited a greater proportion of human collagen, while multi-cellular tumors exhibited a significantly greater quantity of mouse collagen.

As no differences in glucose uptake and hypoxia were observed between PanC-1 and multi-cellular tumors, we hypothesized that HPaSteC gets lost during tumor growth. To test this hypothesis, a marker for PSCs was needed. Fibroblast activation protein alpha (FAPα) is expressed in HPaSteC but is negligible in PanC-1 cells, as confirmed by western blot analysis. FAPI PET tracers bind specifically to the enzymatic domain of FAPα [61] and can visualize tumor stroma formation [62]. In our studies, we used [^18^F]FAPI-74 as a PET tracer, which exhibits favorable properties in terms of longer half-life and better spatial resolution compared to ^68^Ga-based FAPI tracers [63].

Significantly higher [^18^F]FAPI-74 uptake in HPaSteC spheroids compared to PanC-1 spheroids confirmed its suitability for imaging HPaSteCs. Subcutaneously injected HPaSteC did not form tumors, as previously described [48]. However, the injected cells performed show [^18^F]FAPI-74 uptake, which ceased between 3 and 4 weeks after injection, thereby indicating HPaSteC cell death. In contrast, xenograft models of multi-cellular and PanC-1 showed an increase of [^18^F]FAPI-74 uptake, which corresponded to FAPα expression in the tissue. Although α-SMA is typically considered a marker for pancreatic stellate cells [64,65,66], immunohistochemical staining demonstrated that tumors cultured without pancreatic stellate cells were positive for α-SMA. Sequential staining revealed that infiltrating mouse cells are responsible for α-SMA expression in the tumors. Therefore, the increase in [^18^F]FAPI-74 uptake over the course of 7 weeks is attributed to infiltrating mouse cells. Even though there are some publications discussing xenografts of pancreatic stellate cells and pancreatic cancer cells [57,67], there is limited information available on whether these xenografts exhibit similar mouse cell invasion. Biswas et al. described the implantation of V2 rabbit carcinoma cells into nude mice and found that 30% of the collagen in the tumor was of mouse origin [68]. Olsen et al. described mouse fibroblasts invading MCF7S1 breast cancer tumors. The invasion of murine fibroblasts was even higher in co-culture using MCF7S1 with HMF3s human mammary fibroblasts, where human fibroblasts were completely replaced by mouse fibroblasts [69]. Scarlett et al. detected in a mixed-gender model with bone marrow-derived stem cells that during cancer progression, mouse cells were recruited and differentiated into stellate cells [70]. The Banbury Center meeting’s Consensus Statement on the role of cancer-associated fibroblasts (CAFs) concluded that co-cultures with CAFs pose a challenge due to the presence of mouse fibroblasts that can outgrow the co-injected CAFs [16]. This is precisely what we observed in our experiments.

**Figure 7:**
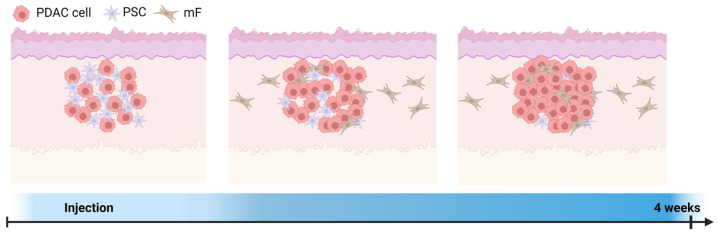
Schematic representation of a subcutaneous PDAC+PSC tumor. Injected tumor cells attract mouse fibroblasts. As the tumor cells divide, more mouse fibroblasts invade the growing tumor mass, and the human pancreatic stellate cells become overgrown. Created in BioRender. Doctor, A. (2024) BioRender.com/k83f778.

The presented results allow us to draw a timeline of the cellular composition in our xenograft models (Figure 7). At the beginning, the human tumor cells proliferate and eventually outgrow the human PSCs during tumor growth. Subsequently, the human PSCs decline after a certain latency period. Simultaneously, host mouse fibroblasts are recruited and migrate into the tumor. Depending on the time frame, the growing tumor represents a mixture of predominantly cancer cells and PSCs, or mouse fibroblasts. This has implications for targeting stromal cells, as potential species differences regarding the molecular target structures need to be considered. Yet, PSCs influence tumor formation and promote the invasion of mouse fibroblasts, as observed by a higher percentage of mouse Collagen I and FAPα and lower NuMA staining. Further, PSCs induced epithelial-mesenchymal transition in multi-cellular tumors as indicated by loss of KRT19 expression, which is also associated with poorer survival and shorter progression-free survival in PDAC patients [71,72,73,74,75]. A limitation of this study is that PDAC is a highly heterogeneous cancer, which can only be inadequately represented by the use of a single PDAC cell line. Consequently, further studies are required to evaluate the impact of other cell lines on the presented models. This research provides valuable insights into tumor-stroma interactions by quantifying the decline of pancreatic stellate cells (PSC) and the invasion of mouse fibroblasts within the tumor microenvironment in vivo, which have been previously described only in limited detail for other tumor entities [66,67,68]. Furthermore, the use of the [^18^F] FAPI-74 radiotracer allows for non-invasive tracking of these interactions, offering a new perspective that has not been extensively explored in a preclinical model in existing literature.

## 5. Conclusions

In conclusion, the spheroid and xenograft models presented here replicate the physiological conditions observed in clinical settings, including impaired vascularization, hypoxia, and stromal components, which are all characteristics of PDAC. However, the proportion of viable PSCs in xenograft tumors declined over time, indicating a necessity to monitor PSC viability in PDAC+PSC models when targeting stromal cells or studying their interaction with tumor cells. Moreover, our findings demonstrate the influence of mouse fibroblasts on the tumor microenvironment of xenografts, thereby contributing to a more precise understanding of their influence on preclinical experiments. This study employed a non-invasive radiotracer setup to assess the reliability of the models and to identify potential avenues for the design of new diagnostic and therapeutic approaches.

## Data Availability

The data presented in this study is contained within the article.

## Supplementary Materials

The following supporting information can be downloaded at: https://www.mdpi.com/article/10.3390/cancers16193393/s1, Figure S1: Radiotracer uptake in monolayer cells; Figure S2: HAP1 and HAP1/PLOD2Knock-out as multi-cellular xenograft tumors with PanC-1; Figure S3: Representative immunohistological images of PanC-1 multi-cellular tumors with HAP1 and HAP1/PLOD2knockout; Figure S4: In vivo uptake of [^18^F]FAPI-74 displayed as tumor to muscle ratio and SUVmean; Figure S5: Representative immunohistological images of PanC-1 and multi-cellular tumors 12 days after cell injection; Figure S6: Western Blot with FAPα; Figure S7: Western Blot with αSMA; Figure S8: Scheme of the radiosynthesis of [^18^F]FAPI-74; Radiosynthesis and Automation of [^18^F]FAPI-74; Figure S9: Optimization of reagent concentration; Figure S10: Dependency of RCC on ^19^F-fluoride concentration at 25 µM FAPI-74 precursor concentration; Figure S11: Schematic representation of the modified radiosynthesizer utilized for [^18^F]FAPI-74 synthesis; Figure S12: Representative radio-HPLC chromatogram for the semi-preparative purification of [^18^F]FAPI-74; Table S1: Antibodies for immunohistochemistry; Table S2: Initial Optimization of Radiosynthesis. References [33,38,76,77,78,79,80,81,82,83,84,85,86,87,88,89,90,91,92] are cited in the Supplementary Materials.
