# Supplementary material for: Combined PET Radiotracer Approach Reveals Insights into Stromal Cell-Induced Metabolic Changes in Pancreatic Cancer In Vitro and In Vivo
Source: Cancers (Basel). 2024 Oct 4;16(19):3393. doi: 10.3390/cancers16193393 (PMC11475921; doi:10.3390/cancers16193393)
Supplement: Supplementary file 1 [file cancers-16-03393-s001.zip › cancers-3175419-supplementary.pdf]

Supporting information

Combined PET radiotracer approach reveals insights into stromal cell-induced metabolic changes in pancreatic cancer in vitro and in vivo

Alina Doctor[1,2], Markus Laube[1], Sebastian Meister[1], Oliver C. Kiss[3], Klaus Kopka[1,2,4,5], Sandra Hauser[1], Jens Pietzsch[1,2,*]

[1] Helmholtz-Zentrum Dresden-Rossendorf, Institute of Radiopharmaceutical Cancer Research, Department of Radiopharmaceutical and Chemical Biology, Bautzner Landstraße 400, 01328 Dresden, Germany
[2] Technische Universität Dresden, School of Science, Faculty of Chemistry and Food Chemistry, Mommsenstraße 4, 01069 Dresden, Germany
[3] Helmholtz-Zentrum Dresden-Rossendorf, Institute of Radiopharmaceutical Cancer Research, Department of Targetry, Target Chemistry and Radiopharmacy, Bautzner Landstraße 400, 01328 Dresden, Germany
[4] National Center for Tumor Diseases (NCT) Dresden, Partner Site Dresden, University Cancer Center (UCC), Fetscherstraße 74, 01307 Dresden, Germany
[5] German Cancer Consortium (DKTK), Partner Site Dresden, Fetscherstraße 74, 01307 Dresden Germany

* Correspondence: j.pietzsch@hzdr.de

Contents

1. Antibodies used for western blot and immunohistochemistry 2

2. In vitro radiotracer uptake in monolayer 2

2.1. Method 2

2.2. Results and discussion 3

3. Characterization of HAP1 and HAP1/PLOD2knock-out coculture models with PanC-1 4

4. SUVmean and tumor to muscle ratio of [^{18}F]FAPI-74 in PanC-1 and multi-cellular xenograft tumors 6

5. Immunohistological stainings 7

6. Western Blots 8

7. Radiosynthesis and Optimization of [^{18}F]FAPI-74 9

7.1 Materials and Methods 9

7.1.1. Optimization 10

7.1.2. Automated Radiosynthesis of [18F]FAPI-74 11

7.1.3. 11

7.2. Results 11

References 18

1. Antibodies used for western blot and immunohistochemistry

Table S1: Antibodies for immunohistochemistry

antibody	company	catalogue number	host species	reactivity
**α-SMA**	Abcam	ab150301	rabbit	human, mouse, (rat)
**collagen I**	Abcam	ab138492	rabbit	human
**collagen I**	Abcam	ab270993	rabbit	mouse, (rat)
**FAPα**	Abcam	ab207178	rabbit	human
**FAPα**	LSBio	LS-C831501	rabbit	mouse
**NuMa**	Abcam	ab97585	rabbit	human
**KRT19**	Abcam	ab52625	rabbit	human, mouse
**rabbit IgG**	Abcam	ab37413	rabbit	
**goat anti-rabbit**	Dianova	111-065-003	goat	rabbit

2. In vitro radiotracer uptake in monolayer

2.1. Method

The in vitro radiotracer uptake in monolayers was conducted in accordance with the previously described methodology for spheroids. Following a 30-minute incubation on ice, the cells were lysed using a solution of sodium hydroxide and sodium dodecyl sulfate (NaOH/SDS). The cell lysate was then quantified in a gamma counter. The results were normalized to the protein content, which was determined by BCA.

2.2. *Results and discussion*

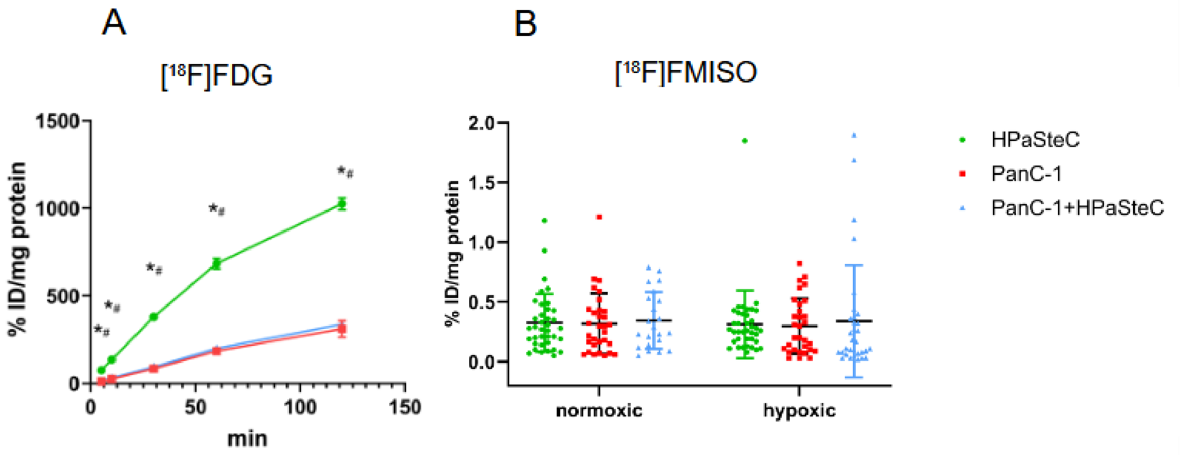

Figure S1: Radiotracer uptake in monolayer cells. (A) In vitro radiotracer uptake assay with HPaSteC, PanC-1 and co-cultured monolayer. Percentage of injected dose per µg (mean+SD) and statistic difference *: HPaSteC vs. PanC-1, #: HPaSteC vs. co-culture ($p<0.05$, two-way ANOVA). (B) time-dependent [^{18}F]FDG uptake. C: [^{18}F]FMISO uptake in normoxic and hypoxic conditions after 4 hours of incubation.

Similar to spheroids (see Figure 2), HPaSteC shows the highest [^{18}F]FDG uptake among the three monolayer cultures. PanC-1 and the coculture exhibit the same size-dependent uptake behavior and radiotracer uptake. No differences in [^{18}F]FMISO uptake are observed among the cultures, regardless of atmospheric conditions.

The data corresponds to that obtained with spheroids. In coculture, the metabolic characteristics shift towards the PanC-1 cells despite the fact that HPaSteC cells are more metabolically active than the PanC-1 cells and are present in larger amounts. No differences in [^{18}F]FMISO uptake were observed between normoxic and hypoxic conditions. We concluded, that the short amount of time on normoxic conditions for washing and inducing lysis is sufficient to reverse the covalent bound and release [^{18}F]FMISO.

3. Characterization of HAP1 and HAP1/PLOD2knock-out coculture models with PanC-1

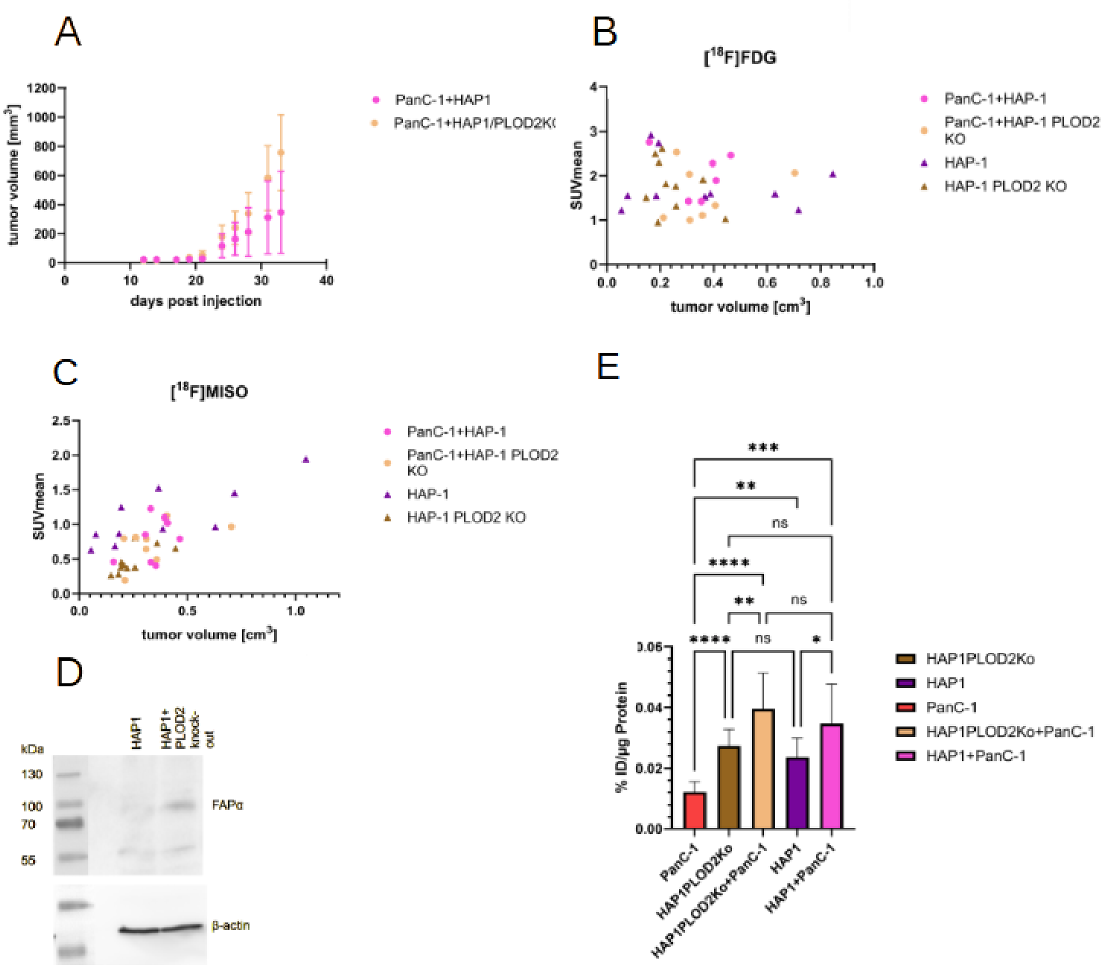

Figure S2: HAP1 and HAP1/PLOD2Knock-out as multi-cellular xenograft tumors with PanC-1 (A) Growth curve of PanC-1 multi-cellular tumors with HAP1 and HAP1/PLOD2Knock-out. Tracer uptake in vivo xenograft tumors (B) [18F]FDG uptake (C) [18F]FMISO uptake (D) FAPα expression was determined via Western blot using spheroid cell lysates. (E) [18F]FAPI-74 uptake in spheroids measured in %ID per µg protein and significance analysis with two-way ANOVA ($p<0.05$, * 0.0332, ** 0.0021, *** 0.0002, **** <0.0001).)

HAP1 cells exhibit a fibroblast-like phenotype and a near-haploid character and are derived from chronic myeloid leukemia. PLOD2 mediates the hydroxylation of lysyl residues on collagen-like peptides. The resulting hydroxylysyl groups serve as attachment sites for carbohydrates in collagen. These carbohydrates are critical for the stability of intermolecular cross-links [1]. The *PLOD2* gene is overexpressed in various cancer diseases and is connected to poor prognosis. Lysyl hydroxylases 2 (LH2), encoded by the *PLOD2* gene, are the key enzymes that mediate the formation of stabilized collagen cross-links. We investigated the effect of *PLOD2* gene knock-out in HAP1 cells on xenograft characteristics [2]. Interestingly, PanC-1+HAP1/PLOD2 knock-out tumors grew faster than the corresponding PanC-1+HAP1 tumors (see Figure S2A). They had a shorter retention time of about 11 days and grew faster compared to the PanC-1 and multi-cellular tumors. Similar to the PanC-1 and PSC coculture tumors imaged with PET/CT, HAP1 and HAP1/PLOD2 knock-out, as well as their respective cocultures, exhibit conforming [18F]FDG uptake and a size-dependent increase in [18F]FMISO.

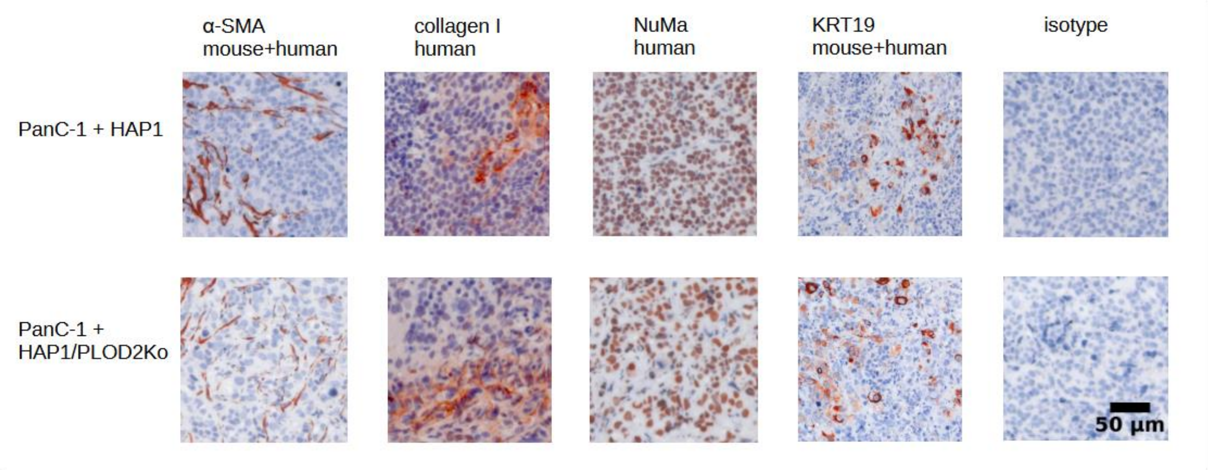

Figure S3: Representative immunohistological images of PanC-1 multi-cellular tumors with HAP1 and HAP1/PLOD2knockout. Staining of markers for α-SMA, human and murine Collagen I, human nuclear mitotic antigen (NuMa), and Cytokeratin 19 (KRT19). Staining control was performed using rabbit isotype antibody. Hematoxylin counter-stains the cell nuclei in blue and positive immunohistological staining is red.

Figure S3 shows immunohistochemical staining of tumor slides with antibodies against α-SMA, human Collagen I, NuMa and KRT19. Both coculture tumors are positive for α-SMA and human collagen I staining. Similar to the immunohistological staining of PanC-1 and multi-cellular (Figure 5), we observe blue nuclei when staining human nuclei (NuMA). This indicates the presence of mouse cells in the xenograft tumor. The invasion into the xenograft tumors in consequently independent of the type of coculture. Moreover, KRT19 staining was observed in PanC-1+HAP1 as well as in PanC-1+HAP1/PLOD2knock-out tumor sections.

4. SUVmean and tumor to muscle ratio of [18F]FAPI-74 in PanC-1 and multi-cellular xenograft tumors

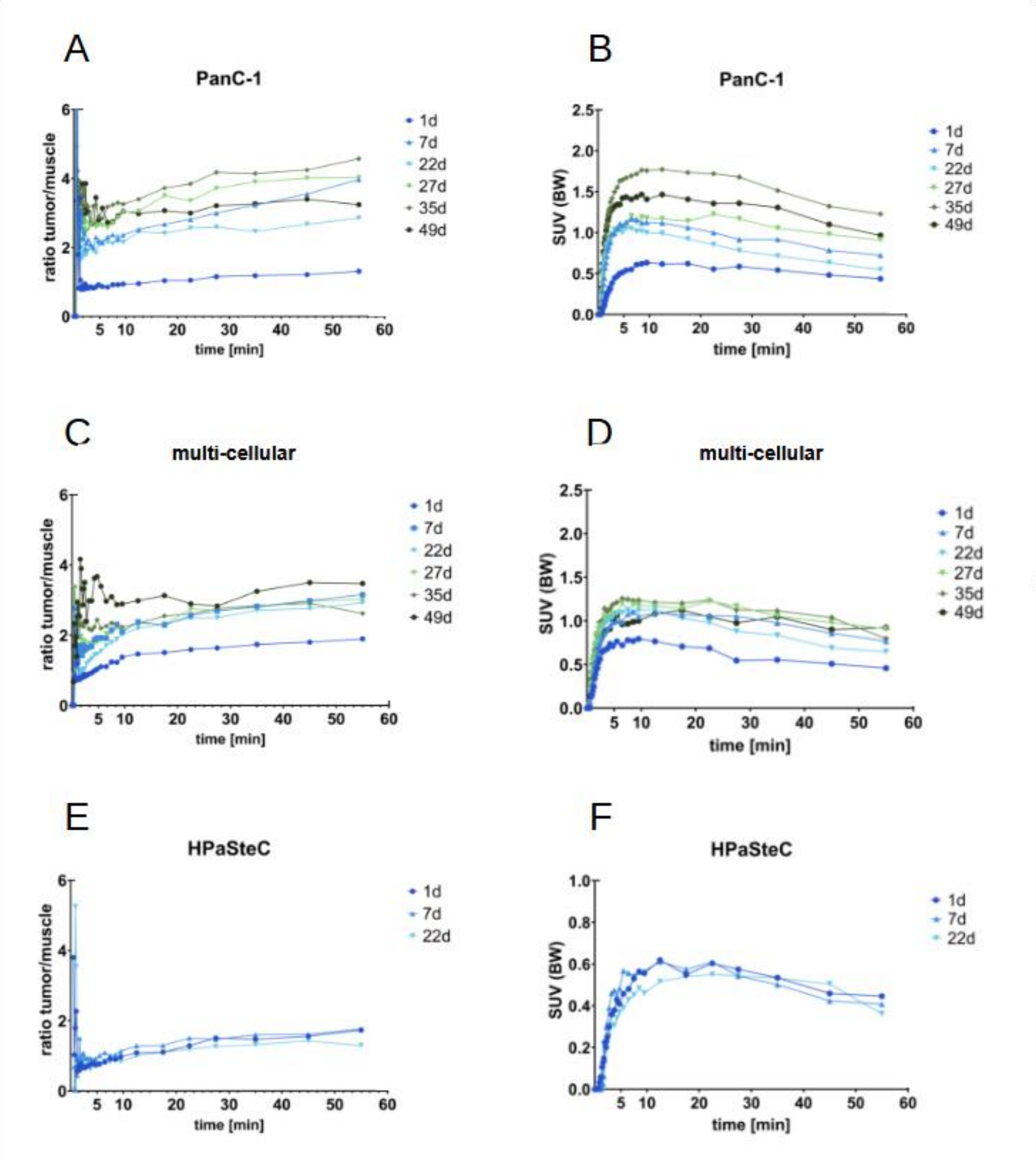

Figure S4: In vivo uptake of [18F]FAPI-74 displayed as tumor to muscle ratio and SUVmean in PanC-1, HPaSteC and coculture xenograft tumors (A+C+E) time dependent tumor to muscle ratio of [18F]FAPI-74 and (B+D+F) time dependent [18F]FAPI-74 uptake (mean values) in the PanC-1 tumor (A+B), the coculture tumor (C+D) or the HPaSteC cells (E+F).

Figure S4 shows that the SUVmean of HPaSteC decreases over time and is the same as the muscle value after 22 days as reflected by the tumor to muscle ratio of 1. SUVmean as well as muscle to tumor ratio is increasing in PanC-1 as well as in multi-cellular tumors.

5. Immunohistological stainings

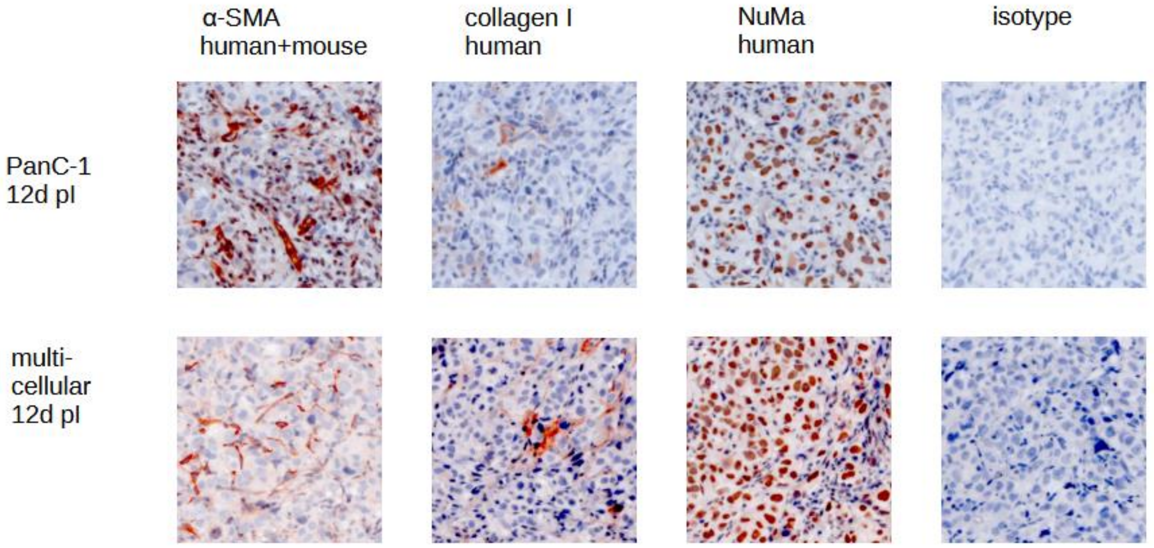

Figure S5: Representative immunohistological images of PanC-1 and multi-cellular tumors 12 days after cell injection. Staining shows α-SMA, human Collagen I and human nuclear mitotic antigen (NuMa). Staining control was performed using rabbit isotype antibody. Hematoxylin as counterstain stains the cell nuclei in blue and positive immunohistological staining is red.

Besides α-SMA staining, Collagen I and NuMa was observed as early as 12 days post-injection. Blue nuclei seen in NuMa staining indicate infiltrated mouse cells.

6. **Western Blots**

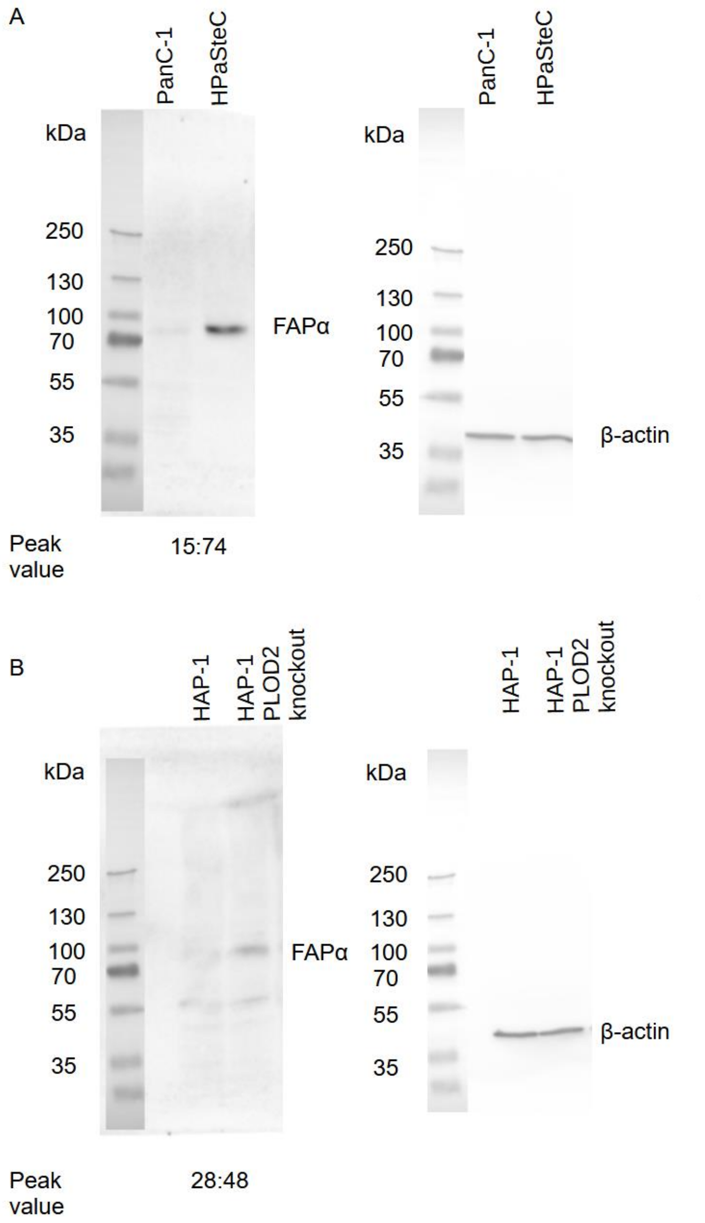

Figure S6: The entire FAPα Western blot image is presented here (A) Western blot of HPaSteC and PanC-1. (B) Western blot of HAP1 and HAP1PLOD2knockout. Both blots with corresponding β-actin and peak value.

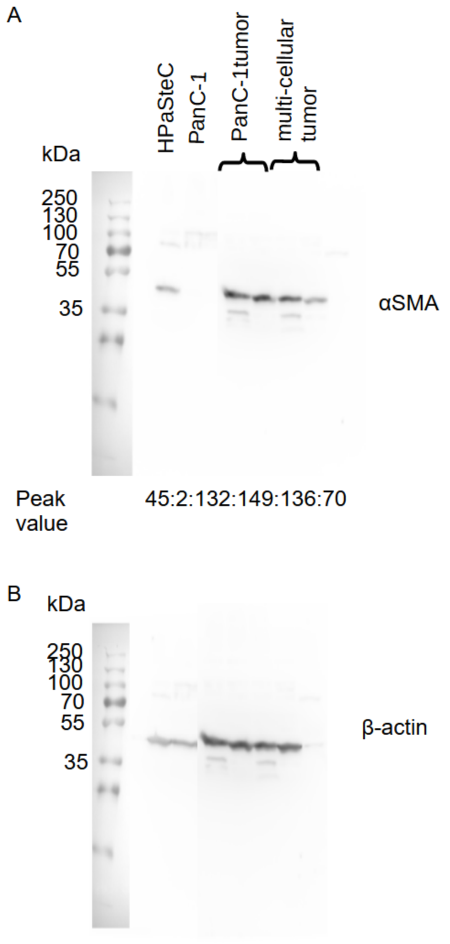

Figure S7: The entire αSMA Western blot image is presented here (A) Western blot of HPaSteC, PanC-1 and tumor lysate of PanC-1 and coculture tumor with peak values. (B) The corresponding β-actin blot is shown.

7. Radiosynthesis and Optimization of $[^{18}F]$FAPI-74

7.1 Materials and Methods

All commercial reagents and solvents were used without further purification. FAPI-74 precursor and FAPI-74 reference standard were obtained from SOFIE (SOFIE Biosciences, Dulles, VA, USA). Stock solutions of aluminum chloride (10 mM in 1 M NaOAc buffer pH 4.0) and 1 M NaOAc buffer (pH 4.0) were stored at 4°C. FAPI-74 precursor was dissolved in DMSO at a concentration of 1 mg/mL (1.256 mM) and aliquots were stored at -20°C.

No-carrier-added aqueous $[^{18}F]$fluoride was produced in a TR-FLEX 18-30 MeV cyclotron (ACSI, Richmond/Vancouver, Canada) by irradiation of $[^{18}O]H_2O$ via the $^{18}O(p,n)^{18}F$ nuclear reaction. Automated radiosyntheses were performed in a hot cell using a radiosynthesizer (Tracerlab FX-N FDG Dual) equipped with a semi-preparative HPLC system: (System 1) semi-preparative C18 precolumn & column Discovery HS F5-5 (5 µm, 250 x 10 mm, Supelco®), JASCO® system: pump PU-980, degasser DG-1580-53, gradient unit LG-980-02 and UV-detector Knaur K2001; MeCN + 0.1%TFA / H_2O + 0.1% TFA 10/90 (v/v), 4 mL/min flow rate; column purging: gradient 10/90 → 95/5 →10/90. The products were monitored at λ = 254 nm and with a radioactivity detector integrated in the synthesizer module. Solid phase extraction was performed using Sep-Pak light Accell Plus QMA® (130 mg, Part. Nr. WAT023525) and Oasis HLB Plus Short (225 mg, Part Nr. 186000132) cartridges.

Analytical HPLC was performed with the following systems: (*System 2*) column Aquity UPLC® HSS T3 column (waters, 100 x 2.1 mm, 1.8 µm, 100 Å) with guard column, UPLC *H*-Class (waters, Milford, Massachusetts, USA): quaternary gradient pump QSM, autosampler FTN, column heater CHA, and diode array detector PDAeλ, γ detector Gabi Star (Raytest), eluent: (A): MeCN / (B): 0.1% trifluoroacetic acid in H_2O; flow rate 0.5 mL/min, gradient: $t_{0\ min}$ 8/92 – $t_{5.0\ min}$ 8/92 - $t_{5.5\ min}$ 95/5 - $t_{6.5\ min}$ 95/5 – $t_{6.8\ min}$ 8/92 – $t_{8.0\ min}$ 8/92; (*System 3*) column Luna C18 (Phenomenex, 250 x 4.6 mm, 5 µm) with guard column, Agilent 1200 HPLC: pump G1311A, auto sampler G1329A, column oven G1316A, degasser G1322A, UV detector G1315D, γ detector Gabi Star (Raytest), flow rate = 1 mL/min, (A) MeCN / (B) H_2O + 0.1% TFA, method 10 iso: $t_{0\ min}$ 10/90 – $t_{10.0\ min}$ 10/90 – $t_{11.0\ min}$ 95/5 – $t_{16.0\ min}$ 95/5 – $t_{17.0\ min}$ 10/90 – $t_{22.0\ min}$ 10/90 or gradient 10→40 $t_{0\ min}$ 10/90 – $t_{10.0\ min}$ 40/90 – $t_{11.0\ min}$ 95/5 – $t_{16.0\ min}$ 95/5 – $t_{17.0\ min}$ 10/90 – $t_{22.0\ min}$ 10/90

Radio-thin layer chromatography (Radio-TLC) was performed on iTLC-SG (Varian, 1.05554.0001). Visualization was performed using a CR35bio scanner system (Raytest, Straubenhardt, Germany) and analyzed using advanced image data analyzer (AIDA) software (Version 5.1 SP4, Raytest, Straubenhardt, Germany).

7.1.1. Optimization

Initial radiolabeling experiments have been performed with a microliter scale approach using HPLC vials [3]. [^{18}F]Fluoride trapping and elution from anion exchange SPE cartridges was performed as indicated. 50 µL eluate were mixed with 2.5 µL 10 mM $AlCl_3$ (25 nmol) and 20 µL FAPI-74 Precursor (25 nmol), and 0, 30, or 80 µL of DMSO with or without premixing of $AlCl_3$ and [^{18}F]fluoride, reaction was carried out at the given temperature for 20 min, and the reaction mixture was analyzed without further dilution (System 2)

Reagent concentration and temperature - Diluted solutions were prepared immediately before the experiment. Aluminium chloride (6.66 mM in 1 M NaOAc pH = 4) was diluted by serial 1:1 dilution with 1 M NaOAc (pH = 4). For each dilution step, DMSO (80 µL) and diluted aluminium chloride solution (2.5 µL) was mixed. FAPI-74 (753 µM in DMSO) was diluted by serial 1:1 dilution with DMSO. For radiolabelling optimization, [^{18}F]fluoride (100-200 MBq) was trapped on a QMA light cartridge and eluted with 0.6-0.8 mL 0.9% saline. For every dilution step, an 50 µL aliquot of [^{18}F]fluoride in 0.9% saline was mixed with 82.5 µL of the diluted aluminium chloride solution in an HPLC vial and the mixture was allowed to stand at room temperature for 5 min. Then, 20 µL of the diluted FAPI-74 precursor solution was added resulting in reaction mixtures containing aluminium chloride and FAPI-74 precursor in a 1:1 ratio at a final concentration of 100 µM, 50 µM, 25 µM, 12.5 µM, 6 µM, 3 µM, and 1 µM, respectively. The sealed vials were heated for 20 min at the indicated temperature. After cooling, the crude reaction mixture was analyzed by radio-HPLC (System 2).

Fluoride concentration - Diluted solutions were prepared immediately before the experiment. 25 µL of aluminium chloride (1.525 mM in 1 M NaOAc pH = 4) was mixed with 200 µL DMSO to give aluminium chloride (169.4 µM in NaOAc/DMSO). 30 µL of FAPI-74 precursor (1.256 mM in DMSO) was diluted with 770 µL DMSO to give FAPI-74 precursor (47.1 µM in DMSO). For radiolabelling optimization, [^{18}F]fluoride (100-200 MBq) was trapped on a QMA light cartridge and eluted with 0.8 mL 0.9% saline. For the highest fluoride concentration, a 90 µL aliquot of [^{18}F]fluoride in 0.9% saline was mixed with 10 µL of a potassium fluoride stock solution (6.863 mM in 0.9% saline). Then 33.3 µL of the ^{19}F-spiked eluate was diluted by serial 1:2 dilution using 66.6 µL aliquots of [^{18}F]fluoride in 0.9% saline. For every experiment, 50 µL of the respective ^{19}F-spiked [^{18}F]fluoride solution was mixed with 22.5 µL aluminium

chloride (169.4 µM in NaOAc/DMSO, 3.81 nmol) in an HPLC vial and the mixture was allowed to stand at room temperature for 5 min. Then, 80 µL of the FAPI-74 precursor (47.1 µM in DMSO, 3.79 nmol) was added resulting in reaction mixtures containing aluminium chloride and FAPI-74 precursor in a 1:1 ratio at a final concentration of 25 µM and varying potassium fluoride concentrations at 0.1 µM, 0.3 µM, 0.9 µM, 2.8 µM, 8.3 µM, 25 µM, 75 µM, and 225 µM. The sealed vials were heated for 20 min at 80°C. After cooling, the crude reaction mixture was analyzed by radio-HPLC (System 2).

7.1.2. Automated Radiosynthesis of [18F]FAPI-74

Preparation – A mixture of 3.04 mM aluminum chloride in 1 M NaOAc pH 4 (25 µL, 76 nmol) and DMSO (200 µL) was placed in the reaction vessel of the radiosynthesizer and the vessel was closed. Saline (0.9%; 0.5 mL, Vial 1), FAPI-74-precursor (95.1 µM in dry DMSO, 800 µL, 76 nmol, Vial 2), 1 M NaOAc pH 4 (3 mL, Vial 4), 0.9% saline (8 mL, Vial 6), water (5 mL, Vial 10), and EtOH (2 mL, Vial 11) was inserted into the reagent vessels. The HPLC collect vial was filled with 30 mL water. All reagents used for the complex formation were handled with plastic or glass ware to reduce metal contaminations in the synthetic process. Sep-Pak light Accell Plus QMA® cartridge was washed with water (5 mL), connected to the radiosynthesizer, and placed in a dose calibrator. Oasis HLB Plus Short cartridge was conditioned with EtOH (10 mL) and water (10 mL) using an ÄKTA prime device. The cooling trap was filled with liquid nitrogen and a 10 L waste gas bag was connected to the exhaust of the radiosynthesizer. The HPLC column was purged and equilibrated at a flow of 4 mL using an eluent of 0.1% TFA in MeCN / 0.1% TFA in water 10/90.

Radiosynthesis - [^{18}F]Fluoride was trapped on a QMA light cartridge and activity was measured (A_{start}). Then, [^{18}F]fluoride was eluted from the QMA light cartridge with saline (Vial 1) and transferred to the reaction vessel which was prepared prior start of synthesis with aluminum chloride in a mixture of 1 M NaOAC/DMSO. The reaction mixture was stirred at room temperature for 5 min for formation of the [^{18}F]aluminum fluoride complex. The mixture was heated to 80°C, allowed to react at this temperature for 10 min and then cooled to 45°C. After [^{18}F]FAPI-74 formation, the reaction mixture was diluted with 1 M NaOAc pH 4 (vial 4), stirred for 1 min and the reaction mixture was further purified by semi-preparative HPLC (System 1, t_R at ~15 min). The product containing fraction was collected, diluted with water (30 mL) and passed through an HLB Plus Short cartridge. The SPE cartridge was washed with water (Vial 10). [^{18}F]FAPI-74 was eluted from the SPE with 2 mL EtOH (Vial 11) and transferred to an adjacent hot cell. When product was formulated directly in 20% ethanol in saline, the 0.9% saline was added to the product via Vial 6. Product activity was measured (A_{EOS}) in a dose calibrator and radiochemical purity (RCP) and molar activity (Am) were determined by radio-HPLC (System 3, Method 10 iso) and optionally with radio-TLC.

7.1.3. Stability of [18F]FAPI-74 in EtOH, 10% EtOH in water, and 10% EtOH in saline (0.9%)

[^{18}F]FAPI-74 in EtOH and 10% EtOH in saline (0.9%) was analysed by repetitive HPLC analyses (System 3) of the mixtures over a time course of 2 hours.

7.2. Results

The method of radiolabeling small biomolecules for PET with ^{18}F using the [^{18}F]aluminum fluoride complexed by the NOTA-chelator was first reported more than a decade ago [4]. Since then, several improvements and applications using peptide, protein and small molecule conjugates have been described which are summarized elsewhere in detail [5-8]. Automated radiosyntheses procedures for [^{18}F]AlF labelling in general [9-12] as well as preparation of [^{18}F]FAPI-74 have been reported [13-16].

Herein we describe our initial optimization results as briefly reported elsewhere [17] and in detail report the optimized radiosynthetic procedure for the automated radiosynthesis applied in this work.

Initial labeling with [18F]AlF has been envisaged by complex formation of [18F]fluoride with aluminum chloride in sodium acetate buffer (pH = 4) at room temperature followed by NOTA complexation at elevated temperatures. While general reaction conditions are known for this type of 18F-chemistry, different synthetic protocols are described for the separation of [18F]fluoride from the target water as well as the concentration and ratio for aluminum chloride and NOTA based precursor as well as the amount and kind of co-solvents. In this regard, we started our attempts with the investigation of elution protocols as well as optimization of reagent concentration in manual synthesis using a recently described microliter scale radiofluorination approach (Table S2 and Figure S7). Radiochemical conversion was analyzed from the crude reaction mixture by radio-UPLC.

We started our work with a screen of different fluoride elution protocols cartridges under non-optimized one pot reaction conditions at precursor concentrations of around 320 μM and reaction at 100°C for 20 min. Elution with acetate buffer from QMA light gave low elution efficiency by normal female side elution, while revers elution from the male side as shown in Table S2, entry 1 gave high elution efficiency and 30% radiochemical conversion as a starting point. Using a polystyrene cartridge and normal elution gave a comparable result (entry 2). The elution of a QMA light with saline shown in entry 4 gave high elution efficiency in normal elution mode and high radiochemical conversion of 26-34%.

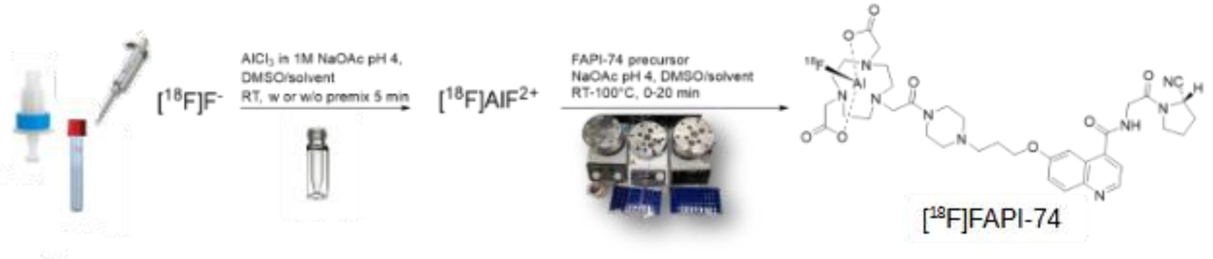

Figure S8: Scheme of the radiosynthesis of [18F]FAPI-74. Reagents and conditions: (a) i: [18F]Fluoride, $AlCl_3$ in 1 M NaOAc pH=4 / DMSO, room temperature, 5 min; ii: FAPI-74 precursor, 60-100°C , 20 min

Table S2: Initial Optimization of Radiosynthesis

#	^{18}F-Trapping & Elution		formation	DMSO concentration	Temperature	RCC (%, HPLC, n=1)
1	0.5 mL 0.5 M NaOAc	QMA SepPak light eluted from revers direction	-	27%	100°C	30
2		10 mg $PSHO_3$	-	27%	100°C	30
3			premix	27%	100°C	36
4	0.5 mL 0.9% NaCl	QMA light	-	27%	100°C	26-34 (n=2)
5			premix	27%	100°C	58
6				66%	100°C	93-100 (n=2)
7					80°C	100
8					40°C	32
9				66% EtOH only	100°C	13

Next, we introduced at this precursor concentration level a premixing time of 5 min for aluminum fluoride complex formation at room temperature which proofed to be beneficial in the acetate buffer shown in entry 3 as well as saline containing system as shown in entry 5. We selected the saline elution protocol for all further experiments due to high elution efficiency and higher radiochemical conversion. It has been demonstrated that the addition of organic solvents like ethanol or DMSO leads to an improvement of the radiolabeling efficiency [18, 19]. A marked increase in reactivity was observed after increasing the DMSO concentration in the final reaction mixture from 27 to 66% leading up to quantitative radiolabeling according to HPLC as given in entry 6. This was still achieved at 80 °C. However, lowering the temperature below 40°C or the use of ethanol instead of DMSO, as shown in entry 8 and 9, were not tolerated by the reaction.

The dependency between radiochemical conversion and reagent concentration as well as temperatures was investigated at a constant 1:1 ratio between aluminum chloride and FAPI-74 precursor as well as constant DMSO and NaOAc amounts (Figure S8A). The reaction temperature of 80°C was found to be optimal and resulted in quantitative radiochemical conversion (RCC) even at concentrations as low as 12.5 µM. At 100°C, only at the highest concentration of 100 µM a quantitative RCC was observed together with steadily decreasing RCC at lower concentrations. Presumably, decomposition of intact FAPI-74 precursor and/or [^{18}F]FAPI-74 as indicated by emergence of a more polar non-radioactive byproduct evident from HPLC analysis is counteracting product formation at this temperature (Figure S8B). Lowering the temperature to 60°C did not improve product formation, quantitative radiochemical conversion was found to occur at a minimal 50 µM reagent concentration. Hence, radiolabeling was performed at 80°C due to optimal balance between favored complex and suppressed byproduct formation.

A

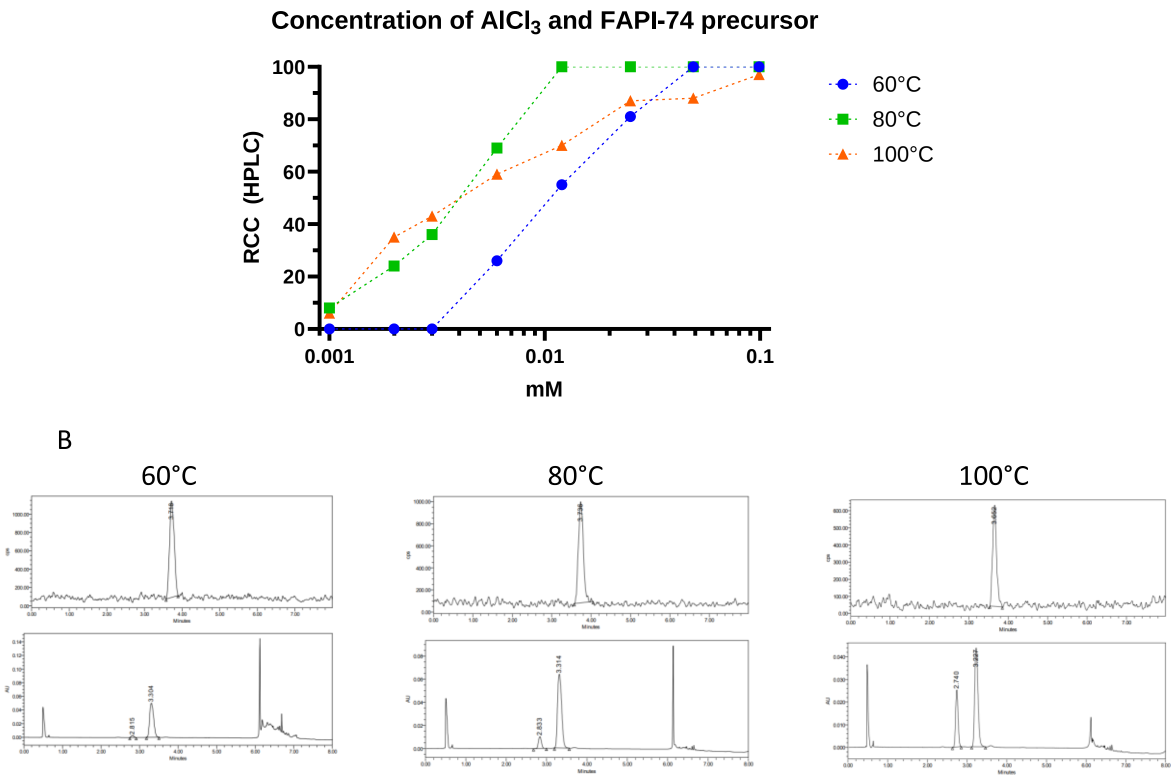

Figure S9: Optimization of reagent concentration. A) Results of radiolabeling at 60°C, 80°C, and 100°C investigated in a concentration range between 1 and 100 µM using $AlCl_3$ and FAPI-74 precursor in a constant 1:1 ratio and analyzed by radio-UHPLC (system 2). B) Representative radio-UHPLC chromatograms of crude reactions mixtures obtained in reactions at 60°C, 80°C and 100°C for 20 min using $AlCl_3$ and FAPI-74 precursor at 100 µM (top: gamma detector, bottom UV at 254 nm). Non-radioactive side product formation with a retention time of 2.8 min is increasing with higher temperature.

The very low concentration suitable for effective radiolabeling with [^{18}F]aluminum fluoride clearly differs from traditional nucleophilic or electrophilic radiofluorinations where the precursor is commonly used in concentrations in the low millimolar range and hence in excess to [^{18}F]fluoride. The molar activity of [^{18}F]fluoride which normally varies in between mid GBq/µmol to low TBq/µmol range hence has to be taken into account. We investigated reactivity under different stoichiometric ratios

between [^{18}F/^{19}F]fluoride and aluminum chloride/FAPI-74 precursor by standard addition of potassium fluoride in 0.9% saline to the [^{18}F]fluoride containing eluate before radiolabeling.

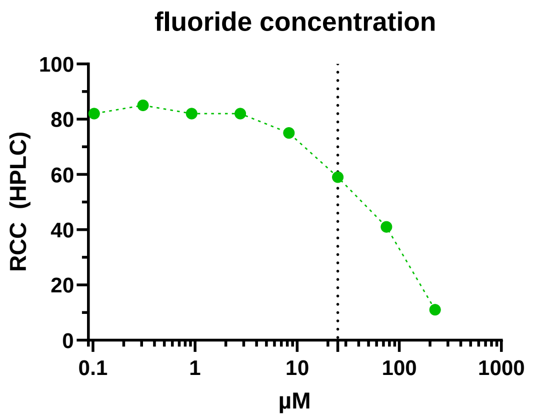

Figure S10: Dependency of RCC on ^{19}F-fluoride concentration at 25 µM FAPI-74 precursor concentration. Radiolabeling was performed at 80°C and a constant $AlCl_3$ and FAPI-74 precursor concentration of 25 µM (dotted vertical line) in the presence of increasing amounts of non-radioactive fluoride (0.1-225 µM).

Applying an aluminum chloride/FAPI-74 precursor concentration of 25 µM, nearly quantitative radiofluorination was observed using up to a fluoride concentration of 8.3 µM which corresponds to a 3-fold excess of FAPI-74 precursor compared to fluoride. By using 1.5 mL reaction volume this corresponds to 12.5 nmol fluoride and hence, at an in-house expected molar activity of 250 GBq/µmol at end-of-bombardment, to a maximal activity amount of 3.2 GBq [^{18}F]fluoride which can be used for quantitative radiolabeling. In turn, we principally adjusted the aluminum chloride and precursor concentration to 50 µM for automated radiosyntheses to obtain [^{18}F]FAPI-74 in suitable activity amounts for preclinical experiments and potentially clinial use but under minimal reagent consumption.

The radiosynthesis was transferred to an automated radiosynthesizer (Figure S10). In brief, [^{18}F]fluoride was trapped on a QMA light cartridge and eluted with saline into the reaction vessel. The vessel contained preloaded aluminium chloride in 1 M acetate buffer pH4 and DMSO to circumvent a reagent addition step and losses due to the low volume of only 225 µL. After a mixing time of 5 min, the FAPI-74 precursor in DMSO was added and the mixture was heated to 80°C for 10 min. The mixture was cooled and diluted with 1 M NaOAc pH4 and further purified by semipreparative HPLC to separate byproducts and non-complexed precursor from the final product (Figure S11, A-B). A final SPE using an HLB cartridge and washing with water followed by elution with ethanol allowed for formulation of the radiotracer in 2 mL EtOH. Identity was proven by coinjection with the non-radioactive reference AlF-FAPI-74 (Figure S11, D-E). Of note, dilution after HPLC in water and washing in water was found to be crucial when a concentration step of the final product was necessary. When residual TFA was present e.g. in initial cases where the dilution and washing step was performed with 0.1% TFA, we obtained a radiochemical pure product. However, during concentration at 70°C under reduced pressure a more hydrophobic by-product was formed in considerable amounts which did not form when TFA was omitted by using water in these last steps. Normally, the product was however obtained in suitable activity concentration and diluted directly in the radiosynthesizer with saline so that no further concentration step was necessary. Of note, in both EtOH and 10% EtOH in saline no sign of degradation of the radiotracer was observed in the time course of 2 hours (Figure S11, F-G).

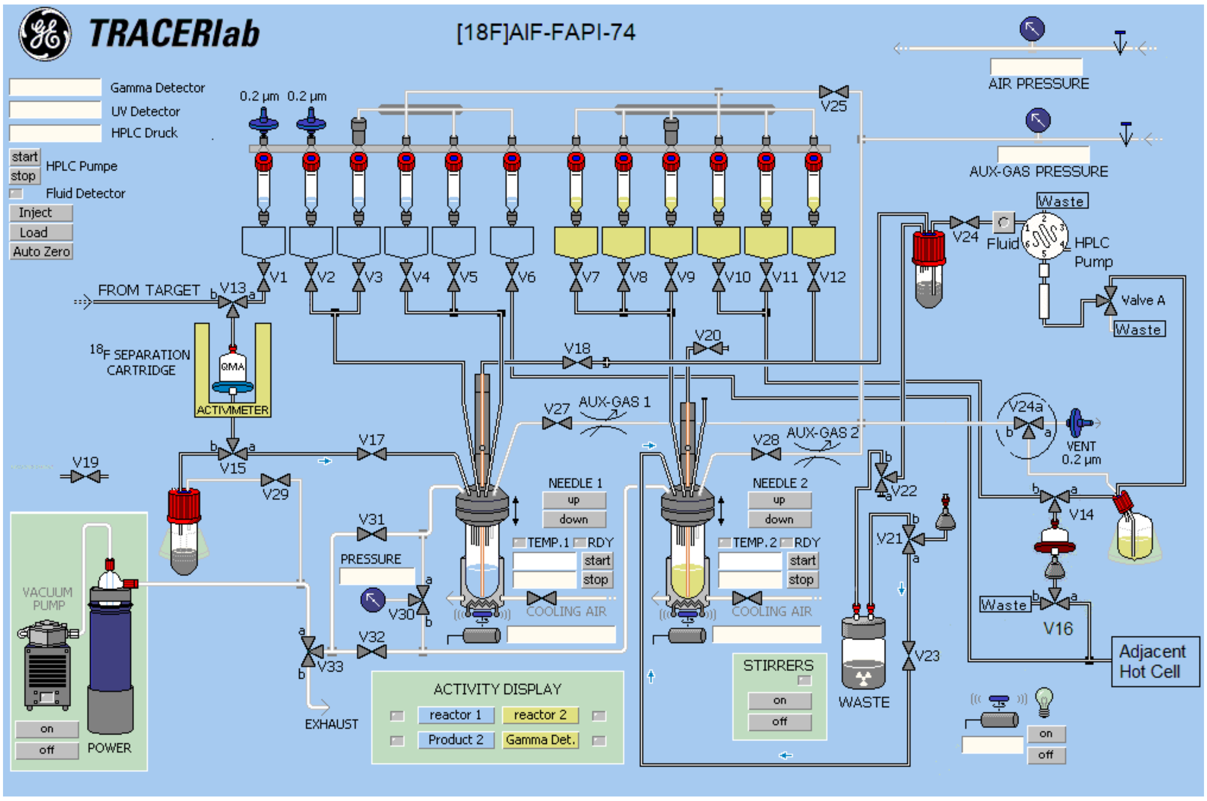

Figure S11: Schematic representation of the modified radiosynthesizer utilized for [^{18}F]FAPI-74 synthesis. Only one reaction vessel was used for the radiosynthesis.

The established protocol allowed for the synthesis of [^{18}F]FAPI-74 with starting activities between 8-145 GBq in 60-70 min total synthesis time furnishing the radiotracer in high radiochemical purity (>98%, radio-HPLC, figure S11-C) and product activity of 2-8 GBq. Radiosyntheses starting from 8-38 GBq at 3-4 hours after bombardment furnished [^{18}F]FAPI-74 in 2-5 GBq and radiochemical yields of 22±7% (n=15) and molar activities 38-203 GBq/µmol at the end-of-synthesis. Interestingly, when starting with considerably higher starting activities of 117-145 GBq directly after the end-of-bombardement we obtained higher isolated product activity of 5-8 GBq and molar activities of 404-689 GBq/µmol as expected but the radiochemical yields were drastically lower (7±2% (n=5)). This principally indicates in accordance with our optimization results, that at higher starting activities also the precursor and aluminum chloride concentration must be increased accordingly when higher product activities are envisaged.

A) Analytical radio-HPLC of the crude reaction obtained in manual syntheses reacting $AlCl_3$ and FAPI-74 precursor at 50 µM at 80°C for 10 min (Method 10 iso)

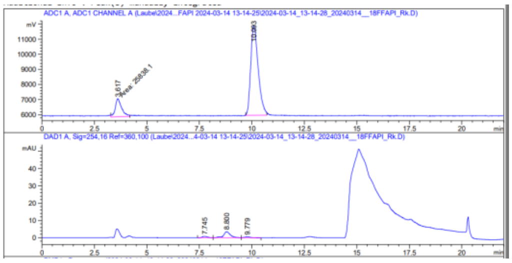

B) Radio-HPLC of semipreparative purification

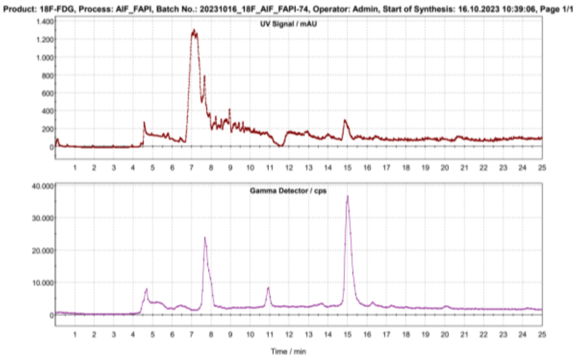

C) Analytical radio-HPLC of the final product (Method 10 iso)

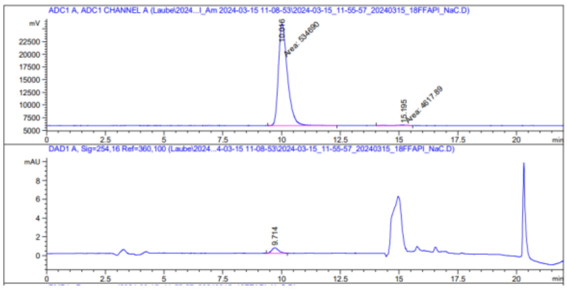

D) Analytical radio-HPLC of the final product (Method 10 grad 40)

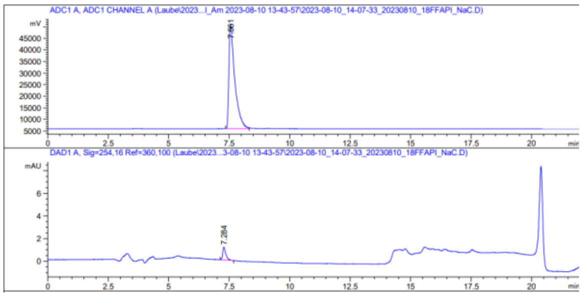

E) Analytical radio-HPLC of the final product coinjected with AlF-FAPI-74 reference (Method 10 grad 40)

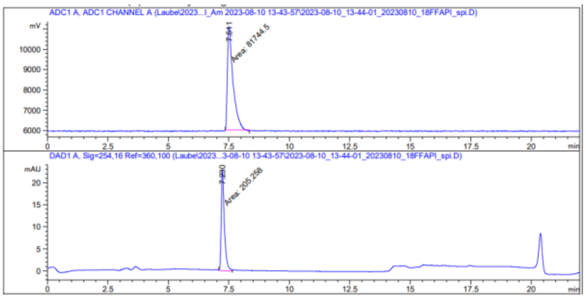

F) Stability in EtOH after 2 h

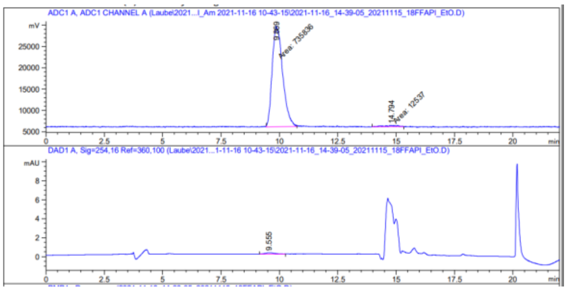

G) Stability in 10% EtOH in saline after 2 h

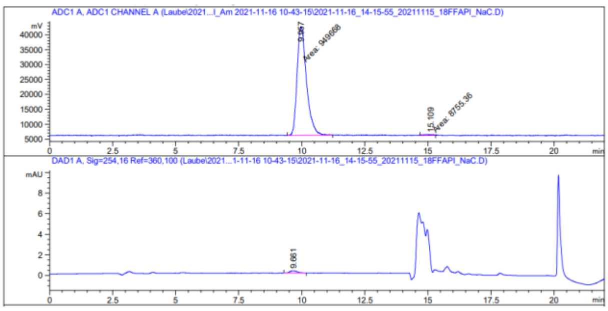

H) Representative radio-TLC of the final product.

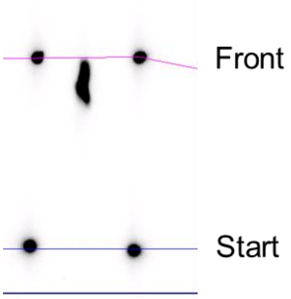

Figure S12: Representative radio-HPLC chromatogram for crude mixture (A, the semi-preparative purification of [^{18}F]FAPI-74 which elutes at ~15 min (B) and analytical data obtained from the final radiotracer (C+D) and final radiotracer coinjected with AlF-FAPI-74 (E) reference as well as the product stored for 2 hours in EtOH (F) and 10% EtOH in saline (G). H: Representative radio-TLC of the final product. Corners of the TLC at the start and front were spotted with ^{18}F activity to allow visualization of the TLC corners during data evaluation.

References

1. Van der Slot, A.J., et al., *Identification of PLOD2 as telopeptide lysyl hydroxylase, an important enzyme in fibrosis.* Journal of Biological Chemistry, 2003. **278**(42): p. 40967-40972.
2. Du, H., et al., *PLOD2 in cancer research.* Biomedicine & Pharmacotherapy, 2017. **90**: p. 670-676.
3. Laube, M., et al., *18F-Chemistry in HPLC vials-a microliter scale radiofluorination approach.* Nuclear Medicine and Biology, 2021. **96**: p. S61.
4. McBride, W.J., et al., *A novel method of 18F radiolabeling for PET.* Journal of nuclear medicine, 2009. **50**(6): p. 991-998.
5. McBride, W.J., R.M. Sharkey, and D.M. Goldenberg, *Radiofluorination using aluminum-fluoride (Al 18 F).* EJNMMI research, 2013. **3**: p. 1-11.
6. Laverman, P., et al., *Al18F labeling of peptides and proteins.* Journal of Labelled Compounds and Radiopharmaceuticals, 2014. **57**(4): p. 219-223.
7. Kumar, K. and A. Ghosh, *18F-AlF labeled peptide and protein conjugates as positron emission tomography imaging pharmaceuticals.* Bioconjugate chemistry, 2018. **29**(4): p. 953-975.
8. Fersing, C., et al., *A comprehensive review of non-covalent radiofluorination approaches using aluminum [18F] fluoride: will [18F] AlF replace 68Ga for metal chelate labeling?* Molecules, 2019. **24**(16): p. 2866.
9. Allott, L., et al., *A general [18 F] AlF radiochemistry procedure on two automated synthesis platforms.* Reaction Chemistry & Engineering, 2017. **2**(1): p. 68-74.
10. Kersemans, K., et al., *Automated radiosynthesis of Al [18F] PSMA-11 for large scale routine use.* Applied Radiation and Isotopes, 2018. **135**: p. 19-27.
11. Giglio, J., et al., *Synthesis of an Al 18 F radiofluorinated GLU-UREA-LYS (AHX)-HBED-CC PSMA ligand in an automated synthesis platform.* EJNMMI Radiopharmacy and Chemistry, 2018. **3**: p. 1-12.
12. Tshibangu, T., et al., *Automated GMP compliant production of [18 F] AlF-NOTA-octreotide.* EJNMMI radiopharmacy and chemistry, 2020. **5**: p. 1-23.
13. Jiang, X., et al., *FAPI-04 PET/CT using [18F] AlF labeling strategy: automatic synthesis, quality control, and in vivo assessment in patient.* Frontiers in Oncology, 2021. **11**: p. 649148.
14. Hu, K., et al., *Preclinical evaluation and pilot clinical study of [18F] AlF-labeled FAPI-tracer for PET imaging of cancer associated fibroblasts.* Acta Pharmaceutica Sinica B, 2022. **12**(2): p. 867-875.
15. Giesel, F.L., et al., *FAPI-74 PET/CT using either 18F-AlF or cold-kit 68Ga labeling: biodistribution, radiation dosimetry, and tumor delineation in lung cancer patients.* Journal of Nuclear Medicine, 2021. **62**(2): p. 201-207.
16. Naka, S., et al., *One-pot and one-step automated radio-synthesis of [18F] AlF-FAPI-74 using a multi purpose synthesizer: a proof-of-concept experiment.* EJNMMI Radiopharmacy and Chemistry, 2021. **6**(1): p. 28.
17. Laube, M., et al. *Optimization and automation of radiolabeling FAPI-74 using [F-18] AlF chemistry*. in *EUROPEAN JOURNAL OF NUCLEAR MEDICINE AND MOLECULAR IMAGING*. 2021. SPRINGER ONE NEW YORK PLAZA, SUITE 4600, NEW YORK, NY, UNITED STATES.
18. D'Souza, C.A., et al., *High-yielding aqueous 18F-labeling of peptides via Al18F chelation.* Bioconjugate chemistry, 2011. **22**(9): p. 1793-1803.
19. Laverman, P., et al., *Optimized labeling of NOTA-conjugated octreotide with F-18.* Tumor Biology, 2012. **33**: p. 427-434.
